# The correlation between characteristics and pharmacological effects of monoterpene glycosides and tannins in Radix Paeoniae Alba

**DOI:** 10.1016/j.jpha.2025.101471

**Published:** 2025-10-25

**Authors:** Qitong Zheng, Mengyao Chen, Jialiang Ying, Zhichao Wang, Qiyuan Shan, Xia-Nan Sang, Gang Cao

**Affiliations:** School of Pharmacy, Zhejiang Chinese Medical University, Hangzhou, 310053, China

**Keywords:** Material basis, Radix Paeoniae Alba, Monoterpene glycosides, Tannins, Pharmacological action

## Abstract

The pharmacodynamic material basis constitutes the central element of traditional Chinese medicine (TCM) in disease treatment. By summarizing the active compounds' characteristics, bioavailability, pharmacological effects, and molecular mechanisms, we can explain the complex interactions between TCMs and diseases. Previous studies have demonstrated that monoterpene glycosides and tannins are related to the pharmacological activity of Radix Paeoniae Alba (RPA). However, research on RPA has primarily focused on monoterpene glycosides, and the functional role of tannins in RPA has received little attention. Observations from animal studies indicate that monoterpene glycosides and tannins exhibit poor bioavailability. Carboxylesterase, produced by gut microbiota, is crucial for metabolizing these compounds in the intestine. Monoterpene glycosides and their gut metabolites can be absorbed into the bloodstream, exerting various pharmacological effects, including anti-inflammatory, immunomodulatory, and neuromodulator activities. In contrast, tannins consist of highly hydrophobic polyphenols that form insoluble protein-tannin complexes. Due to their inability to cross the intestinal barrier, tannins primarily exert localized pharmacological effects within the digestive system. This study systematically reviews the pharmacological activities and mechanisms of monoterpene glycosides and tannins in RPA, while establishing their therapeutic contributions to the herb's pharmacological effects.

## Highlights

• Monoterpene glycosides and tannins are the two main compounds of Radix Paeoniae Alba, which all have poor bioavailability.
• A key intestinal carboxylesterase transformed monoterpene glycosides and tannins.
• Monoterpene glycosides exert anti-inflammatory, immunomodulatory, and neuromodulatory pharmacological effects.
• Tannins have limited bioavailability due to hydrophobicity, focusing their effects on the digestive system.
• Glycosides and tannins' activity are related to Radix Paeoniae Alba's pharmacological effects.

## Graphical abstract

**Figure:**
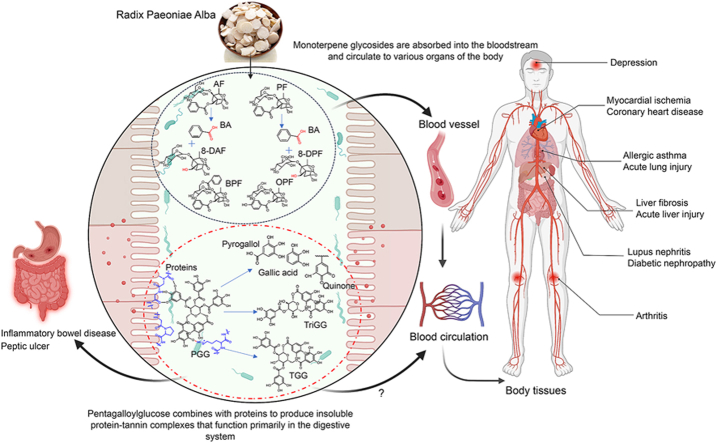

## 1 Introduction

Traditional Chinese medicines (TCMs) offer numerous therapeutic benefits, including minimal side effects, a high safety profile, and abundant availability, making them widely employed in clinical research in recent years. Radix Paeoniae Alba (RPA) has traditionally been used to treat various conditions like pain, depression, inflammation, and immune disorders. Its diverse pharmacological properties and rich content of bioactive compounds enable extensive application in multiple diseases [1]. Current literature shows that RPA's active components hold significant potential in managing gastrointestinal diseases, liver injury, immune disorders, asthma, and other conditions [2]. Thus, a comprehensive review of RPA's molecular mechanisms and pharmacological effects is vital for ensuring safe clinical use, optimizing TCMs formulations containing RPA, and developing new RPA-based drugs and therapies.

RPA is the dried root of *Paeonia lactiflora* Pall., a species of the genus Paeonia, which has received extensive attention in phytochemical analyses. Over the past few decades, Paeonia plants have gained growing prominence due to their medicinal and edible value. Comprehensive chemical composition studies have been conducted and have demonstrated that Paeonia contains numerous bioactive compounds, including monoterpene glycosides, tannins, terpenoids, and steroids, etc. Analyses have revealed that monoterpene glycosides and tannins are predominantly concentrated in the roots and cortex, flavonoids are mainly localized in the flowers, and terpenoids and steroids primarily accumulate in the root cortex and callus tissues [3]. Therefore, monoterpene glycosides and tannins are the two main compounds of RPA because they are derived from the root of *Paeonia lactiflora* Pall. However, most research has focused on monoterpene glycosides due to their unique structures and demonstrated roles in modulating the functions and activation of immune cells and reducing inflammatory mediator production. Our previous studies identified tannins as another major bioactive component in RPA [4]. Although existing research has reported various pharmacological activities of tannins, their specific therapeutic contributions within RPA have been largely overlooked in the current literature. This study comprehensively summarizes the pharmacological effects of the major monoterpene glycosides and tannins in RPA. Furthermore, we systematically analyze the existing literature to elucidate the relationship between RPA's material basis (active components) and its pharmacological effects, particularly focusing on its multimodal pharmacological activities and their potential associations with key bioactive constituents. These findings will offer valuable insights for future RPA research and serve as a reference for investigating the pharmacodynamic material basis of RPA.

## 2 Phytochemical investigations of RPA

Investigating the material basis of TCMs constitutes a fundamental scientific challenge in its inheritance, development, and innovation. RPA, as the dried root, serves as the official medicinal part of *Paeonia lactiflora* Pall. This tissue-specific distribution accounts for the characteristic predominance of monoterpene glycosides and tannins in the fingerprint chromatograms of standardized RPA extracts [5]. And then explains their pharmacological effects as the primary bioactive constituents.

**Fig. 1:**
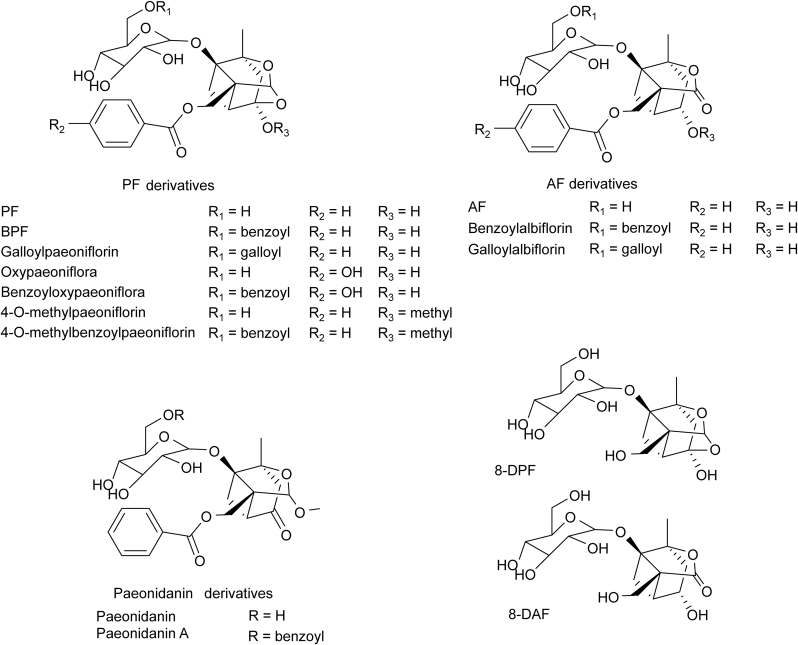
Chemical structures of monoterpene glycosides in Radix Paeoniae Alba (RPA). PF: paeoniflorin; BPF: benzoylpaeoniflorin; AF: albiflorin; 8-DPF: 8-debenzoylpaeoniflorin; 8-DAF: 8-debenzoylalbiflorin.

Terpenes are a class of unsaturated hydrocarbons synthesized from isoprene units, with molecular formulas representing integer multiples of (C_5_H_8_)_n_. As early as 1963, Aimi et al. [6] successfully isolated paeoniflorin (PF), a characteristic monoterpene glycoside, from RPA. Structurally, PF consists of a monoterpene skeleton with benzoyl and glucosyl substituents. Monoterpene glycosides extracted and separated from RPA include PF, albiflorin (AF), oxypaeoniflorin (OPF), benzoylpaeoniflorin (BPF), benzoyloxypaeoniflorin, mudanpioside E, mudanpioside C, mudanpioside H, galloylpaeoniflorin, paeonin D, 4-*O*-ethylpaeoniflorin, benzoylalbiflorin, 8-debenzoylpaeoniflorin (8-DPF) [7], 8-debenzoylalbiflorin (8-DAF), and so on (Fig. 1). The discovery of these monoterpenes provides new options for drug development.

**Fig. 2:**
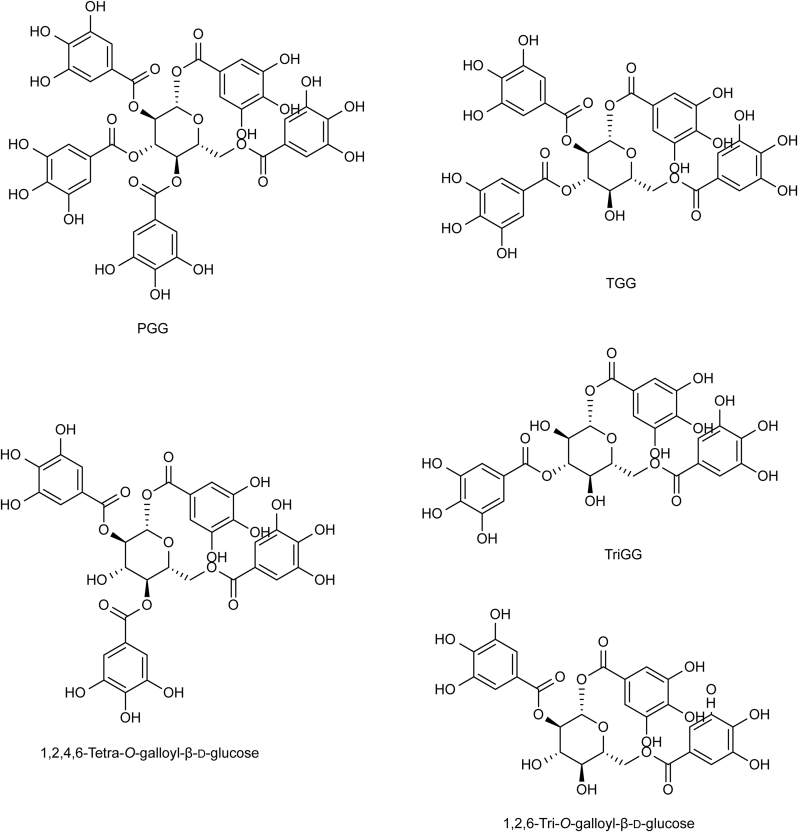
Chemical structures of tannins in Radix Paeoniae Alba (RPA). PCG: 1,2,3,4,6-penta-*O*-galloyl-β-D-glucose; TGG: 1,2,3,6-tetra-*O*-galloyl-β-D-glucose; TriGG: 1,3,6-tri-*O*-galloyl-β-D-glucose.

Tannins are complex polyphenolic compounds in the plant kingdom. The modern definition is a plant polyhydric phenol, formed by mixing the glucose ester of gallic acid and flavanol. Regarding biological activity, tannins exhibit anti-oxidation, anti-tumor, anti-virus, and hemostasis properties [8]. According to their properties and structures, they can be classified as hydrolyzable, condensed, or complex tannins. Tannins isolated from RPA are mainly hydrolyzable tannins, including 1,2,3,4,6-penta-*O*-galloyl-β-D-glucose (PGG), 1,2,3,6-tetra-*O*-galloyl-β-D-glucose (TGG), 1,2,4,6-tetra-*O*-galloyl-β-D-glucose, 1,3,6-tri-*O*-galloyl-β-D-glucose (TriGG), and 1,2,6-tri-*O*-galloyl-β-D-glucose [9]. Our previous study found that PGG has the highest content among tannins in RPA [4] (Fig. 2).

## 3 The bioavailability and degradation of monoterpene glycosides and tannins from RPA

Before reviewing the bioavailability of monoterpene glycosides and tannins, we conducted a comparative analysis of their drug-like properties using the Traditional Chinese Medicine Systems Pharmacology Database and Analysis Platform (TCMSP) database, including oral bioavailability, drug-likeness, and Caco-2 permeability parameters. The drug screening criteria from the TCMSP database were applied as follows: compounds with an oral bioavailability of ≥20% were considered to have sufficient absorption potential, while a drug-likeness score of ≥0.1 was used as the threshold based on pharmacokinetic standards. Additionally, Caco-2 cell permeability was assessed, and high permeability was defined as an apparent permeability coefficient (Papp) value exceeding 1.0 × 10^−5^ cm/s (logPapp > −5.0) [10]. Additionally, we summarized the current literature findings regarding the tissue distribution patterns of these bioactive components (Table 1) [[11], [12], [13], [14]].

**Table 1:** Pharmacokinetic profiling of Radix Paeoniae Alba (RPA)'s bioactive components: oral bioavailability, drug-likeness, Caco-2 permeability, and tissue distribution.

Bioactive compound	Oral bioavailability (%)	Drug-likeness	Caco-2 permeability	Distribution of tissues	Refs.
PF	53.87	0.79	−1.47	Blood, small intestine, heart, liver, spleen, lung, kidneys, brain, thymus, testicles, stomach, pancreas, ovary, skin, muscle bone, and fat	[11,12]
AF	12.09	0.77	−1.54	Blood, small intestine, heart, liver, spleen, lung, kidneys, brain, thymus, testicles, stomach, pancreas, ovary, skin, muscle bone, and fat	[11,12]
BPF	31.27	0.75	−0.69	Blood, heart, liver, spleen, lung, kidneys, stomach, small intestine, large intestine, and testicles	[13]
OPF	21.88	0.78	−1.62	Blood, heart, liver, spleen, lung, kidneys, stomach, brain, thymus, small intestine, large intestine, and testicles	[13]
PGG	3.01	0.20	−3.08	–	[14]

PF: paeoniflorin; AF: albiflorin; BPF: benzoylpaeoniflorin; OPF: oxypaeoniflorin; PGG: 1,2,3,4,6-penta-*O*-galloyl-β-D-glucose.

**Fig. 3:**
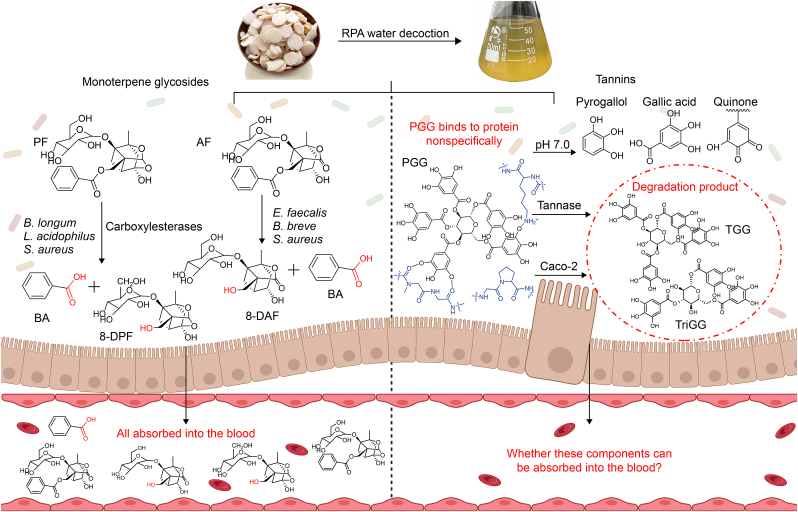
In the gut, *Bifidobacterium longum* (*B. longum*), *Lactobacillus acidophilus* (*L. acidophilus*), and *Staphylococcus aureus* (*S. aureus*) secrete carboxylesterases to convert paeoniflorin (PF) into benzoic acid (BA) and 8-debenzoylpaeoniflorin (8-DPF). Similarly, *Enterococcus faecalis* (*E. faecalis*), *Bifidobacterium breve* (*B. breve*), and *S. aureus* convert albiflorin (AF) to BA and 8-debenzoylalbiflorin (8-DAF). Following oral administration, PF, AF, and their metabolites can be detected in plasma and may contribute to pharmacological activity. In contrast, 1,2,3,4,6-penta-*O*-galloyl-β-D-glucose (PGG) forms insoluble tannin-protein complexes with salivary proteins via hydrogen bonding and hydrophobic interactions, significantly limiting its absorption. At pH 7.0, PGG partially degrades into minor components such as pyrogallol and gallic acid, while the majority is oxidized to form quinones. Additionally, PGG is metabolized by tannase and during Caco-2 transcellular transport, being metabolized into 1,2,3,6-tetra-*O*-galloyl-β-D-glucose (TGG) and 1,3,6-tri-*O*-galloyl-β-D-glucose (TriGG). However, whether these metabolites can be absorbed into the systemic circulation remains unclear.

Monoterpene glycosides exhibited rapid and extensive tissue distribution in all investigated organs [11,12]. However, BPF showed no detectable brain penetration, indicating its inability to cross the blood-brain barrier [13]. Bioavailability studies of RPA's monoterpene glycosides have primarily focused on PF and AF. These compounds demonstrated passive diffusion permeability but had low systemic bioavailability [15,16]. The major metabolic pathway of PF involves hydrolysis by carboxylesterases in both hepatic and intestinal environments, yielding benzoic acid (BA) and 8-DPF as primary metabolites. Gut microbiota studies identified *Bifidobacterium longum* (*B. longum*), *Lactobacillus acidophilus* (*L. acidophilus*), and *Staphylococcus aureus* (*S. aureus*) as the most efficient producers of BA from PF [17]. In this laboratory's previous study on AF metabolism revealed *Enterococcus faecalis* (*E. faecalis*), *Bifidobacterium breve* (*B. breve*), and *S. aureus* as the most active strains in converting AF to BA and 8-DAF [16]. Following oral administration, PF, AF, and their metabolites were detectable in plasma and may contribute to the observed pharmacological effects [18] (Fig. 3).

Numerous tannins have been identified in RPA, with PGG as the prototypical gallotannin. Structurally, PGG consists of five gallic acid molecules esterified to a glucose core, with the β-anomer being the predominant form isolated from plants. Although the TCMSP database reports an oral bioavailability of 3.01% for PGG, experimental studies demonstrate its poor absorption. Li et al. [19] found plasma PGG levels undetectable following high-dose oral administration (80 mg/kg in mice). Similarly, Jiamboonsri et al. [20] reported blood concentrations below the lower limit of quantification (0.039 μM) after administering a 20 mg/kg mixture containing PGG and Methyl gallate. The presence of highly hydrophobic polyphenols in PGG's structure may cause its limited bioavailability. Because TriGG, also a compound of RPA with a few hydrophobic polyphenols and similar structure to PGG, can be detected in rat plasma through the oral route *Turkish galls*, which contain a considerable amount of TriGG [21]. Furthermore, PGG forms insoluble protein complexes in the alimentary canal through interactions with salivary proteins (particularly proline-rich proteins and statherins) via hydrogen bonding and hydrophobic interactions, which likely further restricts its absorption [14].

The pharmacokinetics of tannins remain poorly understood and subject to ongoing debate. While tannins can exert local pharmacological effects in the gastrointestinal tract, systemic bioavailability is required for their broader health benefits. Consequently, careful evaluation of tannin absorption, bioavailability, and metabolism is essential. Current evidence suggests that tannins generally exhibit low bioavailability, and existing research in this area has primarily focused on their degradation pathways. Tannins are degraded under alkaline conditions and by catalytic enzymes from microbes and cells *in vivo*. The hydrolysis of tannin in the alkaline environment is one mechanism of tannin degradation, with polyphenolics undergoing oxidation to form quinones. Tannase also contributes to tannin degradation, and while tannase-producing microbes and cells have been identified, these have so far only been reported in the context of colon cancer [22]. PGG, serving as a model compound for studying tannin degradation, demonstrates instability in alkaline intestinal environments through chemical degradation and poor absorption. At physiological pH (7.0), PGG decomposes into various compounds, including pyrogallol, gallic acid, and lower galloyl glucose esters (TGG and TriGG), while primarily converting to quinones through oxidation [23]. Pharmacokinetic studies revealed only trace amounts of PGG sulfate in blood samples, with no detection in urine or feces [20]. Moreover, *in vitro* and *in vivo* pharmacokinetic studies found the degraded mixture of Methyl gallate and PGG to possibly generate other metabolites similar to gallic acids, including resorcinol, pyrogallol, 4-*O*-methylmalonic acid, pyrogallol-1-*O*-β-D-glucuronide, and 4-*O*-methyl gallic acid-3-*O*-sulfate as downstream products in the intestinal environment. A study showed that PGG underwent degradation during transit across Caco-2 cells, as identifying TGG and TriGG in the receiving compartment suggested the presence of esterase [24] (Fig. 3).

As a result, both monoterpene glycosides and tannins in RPA have poor bioavailability, and both contain ester bonds. These ester bonds are hydrolyzed by intestinal carboxylesterase produced by gut microbiota, leading to the degradation of monoterpenoid glycosides and tannins in the intestine [17]. However, the metabolism and absorption pathways of monoterpenoid glycosides and tannins differ. Monoterpenoid glycosides and their gut metabolites can be absorbed into the bloodstream. In contrast, tannins are composed of highly hydrophobic polyphenols that form insoluble protein-tannin complexes with dietary or mucosal proteins, preventing them from crossing the intestinal barrier. Additionally, there are no reports on whether the downstream products of PGG exert therapeutic effects after translocation into systemic circulation [14]. From a comprehensive perspective, monoterpene glycosides exhibit comparatively higher oral bioavailability than tannins derived from RPA. (Fig. 3).

## 4 Pharmacological effects and molecular mechanisms of monoterpene glycosides from RPA

Monoterpene glycosides represent a class of natural active ingredients in RPA, including PF, AF, BPF, and others. Modern pharmacological studies have demonstrated that monoterpene glycosides derived from RPA exhibit low toxicity, high efficacy, and excellent safety profiles. They possess diverse pharmacological effects, such as anti-cancer, antiviral, cardiovascular protective, neuroprotective, antidepressant, immunomodulatory, and lung injury amelioration properties [25]. Research demonstrates that monoterpene glycosides can reduce the levels of inflammatory factors by inhibiting the mitogen-activated protein kinase (MAPK), phosphatidylinositol 3-kinase (PI3K)/protein kinase B (Akt), NLR family pyrin domain containing 3 (NLRP3), and other inflammatory pathways, thereby exerting anti-inflammatory effects and suppressing the progression of depression, nephritis, hepatitis, and other diseases [26]. Furthermore, recent studies report that RPA-derived monoterpene glycosides show significant anti-tumor activity against various malignancies, including gastric, lung, and liver cancers [27]. The pharmacological effects and mechanisms of total glucosides of paeony (TGP) and monoterpene glycosides are summarized in Table 2 [[28], [29], [30], [31], [32], [33], [34], [35], [36], [37], [38], [39], [40], [41], [42], [43], [44], [45], [46], [47], [48], [49], [50], [51], [52], [53], [54], [55], [56], [57], [58], [59], [60], [61], [62], [63], [64], [65], [66], [67], [68], [69], [70], [71], [72], [73], [74], [75], [76], [77], [78], [79], [80], [81], [82], [83], [84], [85], [86], [87], [88], [89]].

**Table 2:** The pharmacological action of monoterpene glycosides and 1,2,3,4,6-penta-*O*-galloyl-β-D-glucose (PGG) from Radix Paeoniae Alba (RPA).

Natural molecules	Clinical application	Related diseases	Cell/animal model	Dosage	Pharmacological mechanism	Refs.
TGP	Immunological disease	Gouty arthritis	THP-1	2.5, 5, and 10 μg/mL	*MALAT1* and NLRP3↓; *miR-876-5p*↑	[28]
	Kidney disease	AKI	C57BL/6J mice	200 mg/kg	*HCG18* and Bcl-2↑; *miR-16-5p*↓	[29]
			SD rats	50, 100, and 200 mg/kg	*TUG1* and *PTEN*↓; *miR-29a*↑	[30]
	Cardiovascular disease	Myocardial ischemia	Mouse cardiomyocytes HL-1	200 mg/kg	LDH, MDA, ROS, caspase-1, NLRP3, GSDMD-N, and *miR-181a-5*↓	[31]
	Immunological disease	Oral lichen planus	HaCaT keratinocytes	1, 2.5, 5, and 10 μg/mL	IL-6, TNF-α, IκBα, and NF-κB↓	[32]
			Fibroblast-like cells	10 μg/mL	*miR-124*↑; p-STAT3↓	[32]
	Metabolic disease	Diabetes	SD rats	50, 100, and 200 mg/kg	JAK2 and STAT3↓	[33]
	Immunological disease	Lupus nephritis	C57BL/6 mice	100 and 200 mg/kg	p-STAT6 and PD-L2↑	[34]
		Rheumatoid arthritis	DBA/1 mice	0.36 and 0.72 g/kg	NF-κb p65 and STAT3↓	[35]
		Psoriasis	Balb/c mice	180, 360, and 720 mg/kg	STAT1, STAT3, and Th17↓	[36]
			HaCaT cells	10, 50, and 120 mg/L	p-p38 MAPK and NF-κB p65↑; VEGFA and IL-22↓	[36]
		Systemic sclerosis	C57BL/6J mice	100, 200, and 400 mg/kg	IFN↓	[37]
		Arthritis	SD rats	25, 50, and 100 mg/kg	MAPK, PGE2, IL-1, and TNF-α↓	[38]
	Cancer disease	Breast cancer	Polyomavirus middle T antigen mice	400 mg/kg	NF-κB and CCL2↓	[39]
	Cardiovascular disease	Myocardial remodeling	SD rats	100 and 200 mg/kg	NF-κB, MMP-2, and MMP-9↓	[40]
	Liver disease	Drug-induced liver injury	Swiss-derived outbred stock-1 mice	0.234 g/kg	PI3K, MAPK, and NF-κB↓	[41]
		Autoimmune hepatitis	C57BL/6 mice	468, 936, and 1872 mg/kg	Bax, cleaved caspase-3, and DCs↓; Bcl-2↑	[42]
	Nervous system	Parkinson's disease	C57BL/6 mice	0.75, 1.5, and 3 g/kg	Bcl-2, cAMP, and PKA↑; Bax and CREB↓	[43]
	Gastrointestinal disease	Inflammatory bowel disease	C57BL/6 mice	360 and 720 mg/kg	p-Lyn, Snail, TNF-α, IL-17A, IL-23, and IFN-γ↓	[44]
	Mental sickness	Depression	C57BL/6J mice	200 mg/kg	NLRP3, pro-caspase-1, caspase-1, IL-1β, and GSDMD-N↓	[45]
PF	Mental sickness	Depression	C57BL/6 mice	10, 20, and 40 mg/kg	Caspase-11, caspase-1, NLRP3, and IL-1β↓	[46]
			ICR mice	20, 40, and 80 mg/kg	FGF-2 and FGFR1↑	[47]
	Nervous system	Parkinson's disease	C57BL/6 mice	30 mg/kg	JNK and p53↓	[48]
	Immunological disease	Allergic asthma	C57BL/6 mice	1, 5, and 25 mg/kg	NF-κB↓	[49]
		Psoriasis	Hartley guinea pigs	9% PF emulsion	IL-22 and p38 MARK↓	[50]
		Autoimmune encephalomyelitis	C57BL/6 mice	100 μg	Th17, IKK, NF-κB, and JNK↓	[51]
	Metabolic disease	Diabetic foot ulcer	SD rats	15 mg/kg	CXCR2, NLRP3, ASC, and caspase-1↓	[52]
	Gastrointestinal disease	Ulcerative colitis	Balb/c mice	20 mg/kg	NF-κB↓	[53]
	Liver disease	Liver injury	SD rats	0.2, 0.1, and 0.05 g/kg	PI3K and Akt↓	[54]
	Kidney disease	Mesangial proliferative glomerulonephritis	SD rats	50 and 100 mg/kg	PI3K and Akt↓	[55]
	Immunological disease	Lupus nephritis	FasL-deficient B6/gld and C57BL/6 mice	50 and 100 mg/kg	mTNF-α↑; IFN-γ, CXCL1, IL-12, and IL-23↓	[56]
	Genital system	Preeclampsia	HUVECs	10 mmol/L	VEGFA↑; sFlt-1, and sEng↓	[57]
		Deficient endometrial receptivity	C57BL/6 mice	8 mg/kg	LIF↓	[58]
	Cancer	Nasopharyngeal cancer	Human NPC cell lines CNE1/CNE2	20 and 30 μM	NEDD4↓	[59]
		Cancer cachexia	C26/LLC tumor cells	12.5, 25, 50, and 100 μM	TLR4 and NF-κB↓	[60]
		Gastric cancer	MGC-803 cells	0, 5, 10, and 20 μM/L	PI3K and Akt↓	[61]
	Immunological disease	Rheumatoid arthritis	DBA/1 mice	150 mg/kg	IL-6, TNF-α, IL-1β, Th1, and Th17↓	[62]
	Liver disease	Liver injury	C57BL/6 mice	50, 100, and 200 mg/kg	JNK and CYP2E1↓	[63]
			SD rats	20 mg/kg	ROCK, AMPK, and SREBP-1c↓	[64]
	Cardiovascular diseases	Coronary artery disease	HCAECs	0.05, 0.1, 0.2, and 0.4 mM	IL-6, IL-18, and IL-10↓	[65]
			*ApoE*^−/−^ mice	50 mg/kg	β-catenin, c-MYC, and cyclin D1↑; E-cadherin↓	[65]
AF	Nervous system	Neuropathic pain	Wistar rats	50 mg/kg	MAPK↓	[66]
	Mental sickness	Depression	SD rats	15 and 30 mg/kg	5-HT, NE, and DA↑	[67]
		Post-traumatic stress disorder	SD rats	3, 5, 7, and 14 mg/kg	Prefrontal cortex↓	[68]
	Nervous system	Alzheimer's disease	APPswe/PS1dE9 mice	20 and 40 mg/kg	Drp1↓; MFN1, MFN2, and OPA1↑	[69]
	Gastrointestinal diseases	Ulcerative colitis	C57BL/6 mice	50 and 100 mg/kg	AMPK↓	[70]
	Metabolic diseases	Obesity	C57BL/6 mice	5 mg/kg	AMPK, PI3K, and Akt↓	[71]
		Metabolic syndrome	C57BL/6 mice	100 mg/kg	AST, ALT, and TNF-α↓	[72]
BPF	Immunological disease	Anaphylactic diseases	RBL-2H3 cells	5, 25, and 100 μM/L	ERK1/2, JNK, and p38↓	[73]
		Sepsis	C57BL/6 mice	0.09, 0.22, and 0.44 mg/kg	IL-6, TNF-α, IL-1β, and CXCL1↓	[74]
			HUVECS and THP-1-macrophages	2, 5, and 10 μM	p65, JNK, and ERK↓	[74]
	Liver diseases	Fatty liver	L02 and HepG2 cells	2.5, 5, and 10 μM	AMPK↑	[75]
OPF	Cardiovascular disease	Myocardial ischemia-reperfusion injury	BALB/c mice	10, 20, and 40 mg/kg	SIRT1 and Foxo1↑; cTnI and cTnT↓	[76]
	Other	Inflammation	RAW264.7 cells	0, 10, and 30 μM	ERK and p38 MAPK↓	[77]
	Respiratory system	Acute lung injury	C57BL/6 mice	40 mg/kg	PTEN↓; p-Akt↑	[78]
	Metabolic disease	Diabetic nephropathy	Mesangial cells HBZY-1	10 μM	IL-6 and MCP-1↓	[79]
PGG	Virosis	HCV	Huh-7.5 cells, Vero B4 cells, SCID mice, and Balb/c mice	0.5, 1, 2, 4, 8, 16, 20, and 32 μM; 50 mg/kg	Block HCV; reduce liver toxicity	[80]
		Rabies virus	Baby hamster kidney cells	10 μM	*miR-455-5p*, STAT3, and IL-6↓; SOCS3↑	[81]
	Intestinal diseases	Inflammatory bowel disease	C57BL/6 mice	5 and 10 mg/kg	MyD88, NF-κB, TNF-α, IL-1β, and IL-6↓	[82]
	Metabolic disease	Diabetic nephropathy	Mouse mesangial cells	5, 10, and 20 μM	JAK and STAT3↓; Nrf2 and HO-1↑	[83]
	Respiratory system	Emphysema	Primary pulmonary and fibroblasts	100 μg/mL	MMP-12↓	[84]
		Acute lung injury	SD rats	17.16 mg/kg	Neutrophils↓	[85]
			SD rats	15, 30, and 60 mg/kg	AMPK, PI3K, Akt, and Nrf2↑	[86]
	Cancer	Breast cancer	Breast cancer MCF-7 cells	0, 20, 40, 60, 80, and 100 μM	ERBB, PI3K, and Akt↓	[87]
		Liver cancer	SK-HEP-1 cells	30 μM	NF-κB↓	[88]
		Colon cancer	HCT-116 and HT-29 cells	0, 1, 2.5, 5, and 10 μM	p53↑	[89]

TGP: total glucosides of paeony; *MALAT1*: metastasis-associated lung adenocarcinoma transcript 1; NLRP3: NLR family pyrin domain containing 3; *miR-876-5p*: microRNA-876-5p; AKI: acute kidney injury; *HCG18*: human leukocyte antigen complex group 18; Bcl-2: B cell lymphoma 2; SD rats: Sprague-Dawley rats; *TUG1*: taurine upregulated gene 1; *PTEN*: phosphatase and tensin homolog; LDH: lactate dehydrogenase; MDA: malondialdehyde; ROS: reactive oxygen species; GSDMD-N: gasdermin (GSDM) D N-terminal domain; IL-6: interleukin 6; TNF-α: tumor necrosis factor alpha; IκBα: nuclear factor-kappa B (NF-κB) inhibitor alpha; STAT3: signal transducer and activator of transcription 3; JAK2: Janus kinase 2; PD-L2: programmed death ligand-2; Th17: T helper 17; MAPK: mitogen-activated protein kinase; VEGFA: vascular endothelial growth factor A; IL: interleukin; IFN: interferon; PGE2: prostaglandin E2; CCL2: C−C motif chemokine ligand 2; MMP-2: matrix metalloproteinase-2; PI3K: phosphatidylinositol 3-kinase; Bax: Bcl-2-associated X protein; DCs: dendritic cells; cAMP: cyclic adenosine monophosphate; PKA: protein kinase A; CREB: cAMP response element-binding protein; PF: paeoniflorin; FGF-2: fibroblast growth factor-2; FGFR1: FGF receptor 1; JNK: c-Jun N-terminal kinase; IKK: IκB kinase; CXCR2: C−X−C motif chemokine receptor 2; ASC: apoptosis-associated speck-like protein containing a CARD; Akt: protein kinase B; mTNF-α: transmembrane TNF-α; CXCL1: C−X−C motif ligand 1; HUVECs: human umbilical vein endothelial cells; sFlt-1: soluble fms-like tyrosine kinase-1; sEng: soluble endoglin; LIF: leukemia inhibitory factor; NEDD4: neural precursor cell expressed developmentally downregulated protein 4; TLR4: Toll-like receptor 4; CYP2E1: cytochrome P450 2E1; ROCK: Rho-associated coiled-coil kinase; AMPK: AMP-activated protein kinase; SREBP-1c: sterol regulatory element-binding protein 1; HCAECs: human coronary artery endothelial cells; c-MYC: cellular myelocytomatosis oncogene; AF: albiflorin; 5-HT: serotonin; NE: norepinephrine; DA: dopamine; Drp1: dynamin-related protein 1; MFN1: mitofusin 1; OPA1: optic atrophy 1; AST: aspartate transaminase; ALT: alanine transaminase; BPF: benzoylpaeoniflorin; ERK1/2: extracellular signal-regulated kinase 1/2; OPF: oxypaeoniflorin; SIRT1: silent information regulator sirtuin 1; Foxo1: forkhead box protein O1a; cTnI: cardiac troponin I; MCP-1: monocyte chemoattractant protein-1; HCV: hepatitis C virus; SOCS3: suppressor of cytokine signaling 3; MyD88: myeloid differentiation factor 88; Nrf2: nuclear factor erythroid 2-related factor 2; HO-1: heme oxygenase-1; ERBB: erythroblastic leukemia viral oncogene homolog.

### 4.1 TGP

TGP, a complete glycoside derived from RPA, has been clinically approved for managing various autoimmune disorders, including rheumatoid arthritis, lupus, psoriasis, and diabetic kidney disease. Studies show that it has bidirectional immunomodulatory effects on cellular and humoral immunity, as well as inflammatory responses [28]. Beyond immunomodulation, TGP also exhibits anti-stress, hepatoprotective, analgesic, anticoagulant, and cardiovascular-protective properties. This review summarizes TGP's applications, focusing on its gene regulation, signaling pathways, and its pharmacological effects on the immune, hepatic, cardiovascular, and nervous systems.

#### 4.1.1 TGP regulates noncoding RNAs (ncRNAs)

ncRNAs comprise most of the human transcribed genome and play essential roles in various cellular processes. ncRNAs are classified into micro, short, and long ncRNAs (lncRNAs) based on their length. Evidence indicates that lncRNAs can act as microRNA sponges, regulating gene expression [90].

**Fig. 4:**
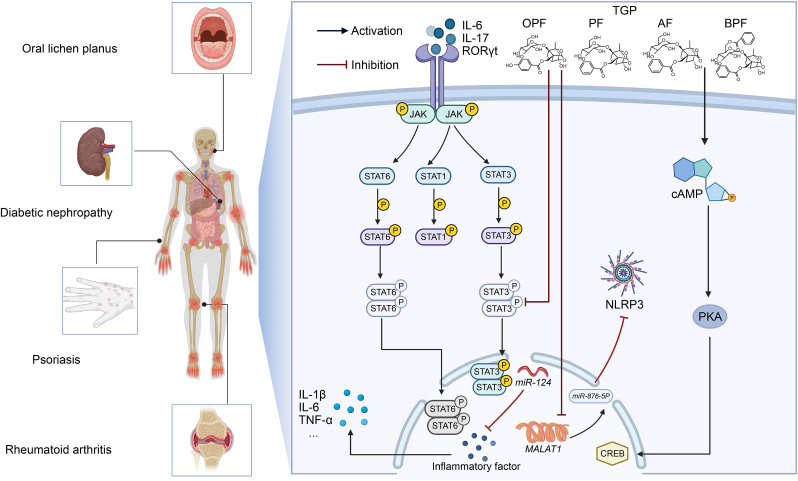
Total glucosides of paeony (TGP) can inhibit the Janus kinase (JAK)/signal transducer and activator of transcription (STAT) pathway, the metastasis-associated lung adenocarcinoma transcript 1 (*MALAT1*)/microRNA-876-5p (*miR-876-5p*)/NLR family pyrin domain containing 3 (NLRP3) pathway, and the cyclic adenosine monophosphate (cAMP)/protein kinase A (PKA)/cAMP response element-binding protein (CREB) pathway, which play crucial roles in the treatment of immune system disorders including oral lichen planus (OLP), psoriasis, diabetic nephropathy, and rheumatoid arthritis. IL-6: interleukin-6; RORγt: retinoic acid receptor-related orphan receptor gamma t; OPF: oxypaeoniflorin; PF: paeoniflorin; AF: albiflorin; BPF: benzoylpaeoniflorin; TNF-α: tumor necrosis factor alpha.

Among the earliest discovered disease-related lncRNAs, metastasis-associated lung adenocarcinoma transcript 1 (*MALAT1*) has been shown to participate in multiple biological processes through its regulatory functions. Extensive evidence demonstrates that *MALAT1* can interact with molecules such as proteins, RNAs, and DNAs, thereby modulating multiple signaling pathways in disease pathogenesis [91]. Meng et al. [28] confirmed the interaction between microRNA-876-5p (*miR-876-5p*) and *MALAT1*, which regulates NLRP3, through RNA immunoprecipitation and RNA pull-down assays. Dysregulation of *miR-876-5p* has been associated with several human cancers. However, the precise role of *miR-876-5p* in gouty arthritis remains unclear. In the human monocytic cell line THP-1 monocytes, TGP attenuates monosodium urate-induced inflammation by regulating the *MALAT1*/*miR-876-5p*/NLRP3 axis (Fig. 4).

##### 4.1.1.1 TGP improves acute kidney injury (AKI) via RNA regulation

Emerging evidence demonstrates lncRNAs' crucial regulatory functions in kidney injury initiation and progression. B cell lymphoma 2 (Bcl-2), an apoptosis-regulatory protein of the Bcl-2 family, mitigates mitochondrial damage and reduces cellular apoptosis in AKI [92]. Research has established the pathogenic role of *miR-16-5p* in septic AKI, while human leukocyte antigen complex group 18 (*HCG18*), which is mainly cytoplasmic, competitively binds *miR-16-5p* [93]. A study demonstrated that TGP alleviates ischemia/reperfusion injury-induced AKI (I/RI-AKI) by upregulating lncRNA *HCG18* to inhibit *miR-16-5p*, thereby promoting Bcl-2 expression and enhancing autophagic activity [29].

Taurine upregulated gene 1 (*TUG1*), a long non-coding RNA, exhibits dysregulated expression patterns across multiple human cancers. Phosphatase and tensin homolog (*PTEN*) serves as a crucial regulator in kidney injury progression through its dual modulation of inflammatory cascades and programmed cell death. Research demonstrates that TGP ameliorates I/RI-AKI by downregulating lncRNA *TUG1* and *PTEN* expression while upregulating *miR-29a* in renal tissue, thereby inhibiting excessive autophagy [30].

Clinical studies have further demonstrated that TGP can effectively treat diabetic nephropathy. The data revealed that after 6 months of treatment, serum levels of high-sensitivity C-reactive protein (hs-CRP), monocyte chemoattractant protein-1 (MCP-1), and tumor necrosis factor alpha (TNF-α) in the TGP group showed significantly greater reductions compared to the losartan group (hs-CRP: *t* = 6.68, *P* < 0.01; MCP-1: *t* = 18.00, *P* < 0.01; and TNF-α: *t* = 8.87, *P* < 0.01). Moreover, prolonged TGP therapy duration was associated with enhanced clinical efficacy, including reduced albuminuria and stabilized renal function [94].

##### 4.1.1.2 TGP reduces cardiomyocyte pyroptosis via the miR-181a-5p/ADCY1 axis

TGP significantly improves cardiomyocyte viability following hypoxia/reoxygenation (H/R) injury while concurrently reducing lactate dehydrogenase (LDH) release, malondialdehyde (MDA) levels, and reactive oxygen species (ROS) production. It increases superoxide dismutase (SOD) activity, suppresses cystathionine γ-lyase activity, and downregulates the expression of NLRP3 and the gasdermin (GSDM) D N-terminal domain (GSDMD-N), along with reducing the secretion of interleukin-18 (IL-18) and IL-1β. Notably, *miR-181a-5p* overexpression or adenylate cyclase 1 (*ADCY1*) inhibition abolished the protective effects of TGP against H/R-induced cardiomyocyte pyroptosis. These results demonstrated that in H/R-treated cardiomyocytes, TGP mitigates pyroptosis through the miR-181a-5p/ADCY1 axis [31].

##### 4.1.1.3 TGP ameliorates oral lichen planus (OLP) through the miR-124/STAT3 pathway

OLP, a common chronic oral mucocutaneous disease mediated by T-cell dysfunction, exhibits significantly enhanced immunomodulatory function in mesenchymal stem cells (MSCs). *miR-124*, a key regulatory microRNA, suppresses inflammatory cytokines by inhibiting nuclear factor-kappa B (NF-κB) signaling. TGP enhances the immunoregulatory capacity of mesenchymal stromal cells via the *miR-124*/signal transducer and activator of transcription 3 (STAT3) signaling pathway while downregulating IL-6 expression (*P* < 0.001) [32] (Fig. 4).

Clinical studies have demonstrated that TGP capsule combined with corticosteroids effectively reduced visual analogue scale scores in patients with reticular OLP (ROLP) and erosive OLP (EOLP), prevented worsening of clinical signs, and showed significantly higher efficacy rates compared to the control group at 1, 3, and 6 months (ROLP: 50% vs. 23.5%, 90.9% vs. 64.7%, and 100% vs. 64.7%; EOLP: 82.4% vs. 64.7%, 88.2% vs. 64.7%, and 100% vs. 88.2%) [95]. These findings further demonstrated that TGP exhibited excellent tolerability with no observed hepatotoxicity, nephrotoxicity, or neurotoxicity, substantiating its profile as a safe and effective therapeutic option for OLP management.

#### 4.1.2 TGP antagonizes the Janus kinase (JAK)/STAT pathway

Tyrosine kinases serve as critical signal transducers, converting extracellular stimuli received through immune and cytokine receptors into intracellular responses. Among these, non-receptor tyrosine kinases of the JAK family play essential roles in various immune-mediated diseases. In unstimulated cells, JAKs are associated with the receptor's cytoplasmic tail. Upon receptor engagement, JAKs undergo phosphorylation and subsequent activation. Following STAT binding to these receptor docking sites, STAT proteins become phosphorylated, leading to their dimerization and nuclear translocation. The active STAT dimers then bind to DNA and modulate transcriptional activity. As key signaling mediators, JAK family members phosphorylate and activate intracellular STAT proteins, which subsequently participate in critical immunomodulatory processes including tumor cell recognition and tumor-driven immune evasion [96] (Fig. 4).

##### 4.1.2.1 TGP plays an essential function in the therapy of kidney damage by blocking the JAK/STAT pathway

Diabetic nephropathy is the leading cause of end-stage renal disease. Activation of the JAK/STAT pathway serves as a key mechanism mediating renal damage under hyperglycemic conditions. In streptozotocin-induced hyperglycemic rats, TGP treatment significantly suppressed the expression of p-JAK2 and p-STAT3 proteins. These results demonstrate that TGP's renal protective effects in diabetic nephropathy are mediated through inhibition of the JAK2/STAT3 signaling pathway [33].

Lupus nephritis, an immune-mediated disorder characterized by macrophage infiltration, can be effectively ameliorated by TGP treatment. In pristane-induced lupus nephritis models, TGP administration reduced urinary protein excretion, serum creatinine levels, and anti-dsDNA antibody titers, while improving renal pathological features and immunological function. Mechanistically, TGP can induce F4/80^+^CD11b^+^PD-L2^+^ and F4/80^+^CD11b^+^CD206^+^ macrophages through the IL-4/STAT6/programmed death ligand 2 (PD-L2) signaling pathway to achieve a therapeutic effect, which may be an effective drug for lupus nephritis clinically in the future [34].

##### 4.1.2.2 TGP alleviates rheumatoid arthritis via the JAK/STAT signaling pathway

TGP demonstrates anti-inflammatory, immunomodulatory, and analgesic properties, making it a widely used therapeutic agent for rheumatoid arthritis in clinical practice. Li et al. [35] demonstrated that TGP treatment alleviated inflammation and reduced joint damage in type II collagen-induced arthritic mice through dose-dependent inhibition of NF-κB p65 and STAT3 phosphorylation.

Clinical study data demonstrated that the incidence of abnormal liver function within 12 weeks was significantly lower in the TGP combination group (TGP plus methotrexate and leflunomide) compared to the control group (methotrexate plus leflunomide alone) (11.38% vs. 23.26%, *P* = 0.013). Additionally, the proportion of patients with alanine transaminase/aspartate transaminase (ALT/AST) levels exceeding three times the upper limit of normal was markedly reduced in the TGP group (1.63% vs. 7.75%, *P* = 0.022). The results indicate that TGP significantly reduces the frequency and intensity of hepatotoxicity induced by combined methotrexate and leflunomide therapy in active rheumatoid arthritis patients, further validating its therapeutic potential for rheumatoid arthritis management [97].

##### 4.1.2.3 TGP ameliorates dermal damage by inhibiting the JAK/STAT signaling pathway

Psoriasis is an inflammatory disorder directly linked to genetic predisposition and autoimmune mechanisms. STAT3 regulates cell cycling and keratinocyte proliferation, which are associated with psoriasis-like skin inflammation. Moreover, decreased STAT3 phosphorylation has been shown to inhibit T helper 17 (Th17) cell differentiation. TGP increased p-STAT3 levels in imiquimod-induced psoriatic mice while suppressing Th17 differentiation and keratinocyte proliferation. Additionally, TGP downregulated p-STAT1, inhibiting Th1 cell accumulation in the epidermis and alleviating dermal inflammatory damage [36].

Systemic sclerosis, distinct from psoriasis, is an autoimmune disease characterized by systemic connective tissue involvement, presenting with skin fibrosis (localized or diffuse), vascular abnormalities, and chronic inflammation. Notably, TGP has been found to attenuate Toll-like receptor 3 (TLR3)-mediated interferon (IFN) responses, thereby blocking the downstream JAK/STAT signaling cascade and ultimately inhibiting scleroderma progression [37].

Moreover, clinical studies have demonstrated that TGP can significantly reduce serum levels of Th1 cytokines, including TNF-α and IFN-γ, in patients with primary Sjögren's syndrome, while also improving xerostomia symptoms. These findings highlight TGP's therapeutic potential in managing immune system disorders [98].

#### 4.1.3 TGP exerts anti-inflammatory effects through modulation of the MAPK signaling pathway

**Fig. 5:**
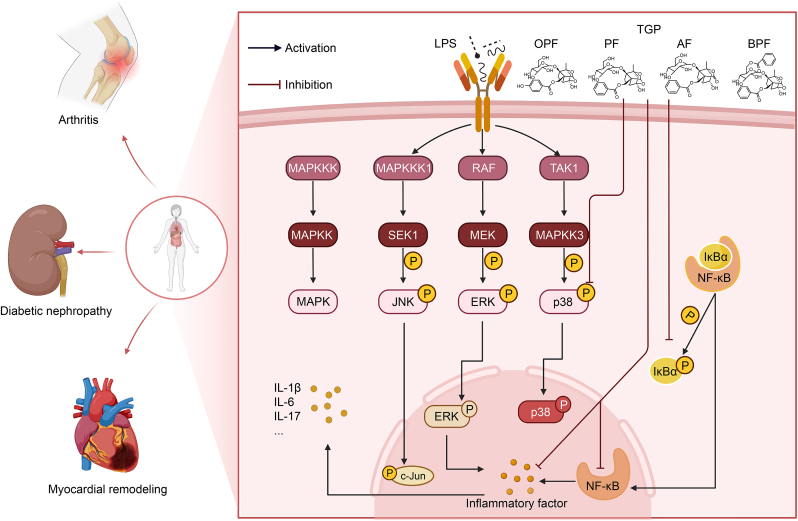
Total glucosides of paeony (TGP) inhibit the phosphorylation of p38 mitogen-activated protein kinase (p38 MAPK) and the activation of nuclear factor-kappa B (NF-κB) signaling pathways, thereby exerting therapeutic effects on arthritis, diabetic nephropathy, and heart diseases. LPS: lipopolysaccharide; OPF: oxypaeoniflorin; PF: paeoniflorin; AF: albiflorin; BPF: benzoylpaeoniflorin; MAPKKK: MAPK kinase (MAPKK) kinase; SEK1: stress-activated protein kinase (SAPK)/extracellular signal-regulated kinase (ERK) kinase-1; JNK: c-Jun N-terminal kinase; RAF: rapidly accelerated fibrosarcoma kinase; MEK: MAPK/ERK kinase; TAK1: transforming growth factor beta (TGF**-**β)-activated kinase 1; IκBα: NF-κB inhibitor alpha; IL-1β: interleukin**-**1β.

MAPK signaling governs key cellular functions including proliferation, differentiation, and migration. The classical MAPK signaling cascade consists of three sequentially acting kinases: MAPK kinase (MAPKK) kinase (MAPKKK), MAPKK, and MAPK. This pathway comprises three major subfamilies: p38 MAPK, extracellular signal-regulated kinase 1/2 (ERK1/2), and c-Jun N-terminal kinase (JNK)/stress-activated protein kinase (SAPK) (Fig. 5). The MAPK cascade (p38, ERK1/2, and JNK) coordinates cell proliferation, differentiation, and stress responses [99].

TGP exerts an anti-inflammatory effect on adjuvant arthritis by modulating the generation of pro-inflammatory mediators in macrophage-like synoviocytes (MLSs) and reducing MAPK phosphorylation in fibroblast-like synoviocytes (FLSs). TGP treatment effectively inhibits the synthesis of prostaglandin E2 (PGE2), IL-1, and TNF-α in MLS and decreases PGE2 levels in complete Freund's adjuvant-induced arthritic rats. Furthermore, TGP demonstrates inhibitory effects on both MAPK activation and matrix metalloproteinase (MMP) synthesis in FLSs exposed to inflammatory supernatants derived from arthritic rats, suggesting suppression of pro-inflammatory mediator-induced signaling cascades [38].

#### 4.1.4 TGP regulates the NF-κB signaling pathway to treat various diseases

NF-κB is indispensable for developing the immune system, inflammation, and innate and adaptive immune responses. In normal cells, NF-κB is typically inactive in the cytoplasm, bound to inhibitor of NF-κB (IκB) proteins, which prevent nuclear translocation. Stimulus-induced IκB degradation permits NF-κB nuclear translocation and target gene transcription. The NF-κB signaling cascade is widely recognized as a canonical regulator of inflammatory responses, principally due to its critical involvement in modulating the transcription of key inflammatory mediators such as intercellular signaling proteins, chemoattractants, and cell surface receptors [100] (Fig. 5).

##### 4.1.4.1 TGP exerts anti-cancer effects via the NF-κB signaling pathway

TGP may suppress tumor immune tolerance to depress breast tumor growth and metastasis by regulating tumor-associated macrophages (TAMs) infiltration and blocking the production of inflammatory factors. Further studies demonstrated that TGP could decrease TAMs' recruitment to promote T cell infiltration in the tumor microenvironment by reducing NF-κB accumulation in the nucleus in macrophages and resulting in suppression of C−C motif chemokine ligand 2 (CCL2), thereby inhibiting 4T1 cell proliferation [39]. Additionally, TGP reduces inflammation by inhibiting the NF-κB signaling cascade in a lipopolysaccharide (LPS)-stimulated HaCaT cell model of OLP [101].

##### 4.1.4.2 TGP suppresses myocardial remodeling by targeting NF-κB signaling

Myocardial remodeling involves pathological fibrosis with excessive extracellular matrix deposition, where cardiac fibroblasts and myofibroblasts form fibrotic scars replacing damaged tissue, ultimately causing chronic heart failure. This remodeling process is closely associated with the NF-κB signaling pathway, which regulates the expression of MMP-2 and MMP-9 messenger RNA (mRNA). TGP has been shown to inhibit MMP-2 and MMP-9 mRNA expression by suppressing NF-κB nuclear translocation, thereby attenuating myocardial remodeling [40].

##### 4.1.4.3 TGP suppresses mammary tumor progression through blockade of the NF-κB-mediated CCL2 signaling axis

Breast cancer is a common malignant tumor in women and the leading cause of cancer death. The CCL2-mediated recruitment of C−C chemokine receptor type 2 (CCR2)-positive monocytes differentiates into TAMs, fostering an immune-tolerant tumor microenvironment with high levels of inflammatory factors. Elevated CCL2 expression not only facilitates TAM accumulation but also contributes to tumor progression [102]. TGP attenuated the immunosuppressive tumor microenvironment by reducing inflammatory mediators and suppressing TAM infiltration, while *in vitro* studies demonstrated its inhibition of LPS-induced IL-10/CCL2 secretion and M2 macrophage polarization, consequently impairing 4T1 cell proliferation. Mechanistic studies revealed that TGP suppressed CCL2 secretion by blocking NF-κB nuclear translocation in macrophages [39].

#### 4.1.5 TGP modulates MAPK/NF-κB pathway for autoimmune disease therapy

Activated MAPK signaling induces NF-κB nuclear translocation, a critical step in the transcriptional activation of inflammatory cytokines (TNF-α and IL-1β) and downstream inflammatory responses [99].

##### 4.1.5.1 TGP mitigates psoriasis progression via MAPK/NF-κB pathway modulation

TGP exhibits therapeutic potential for psoriasis. By inhibiting p-p38 MAPK expression, TGP reduces vascular endothelial growth factor A (VEGFA) and IL-22 levels, thereby alleviating inflammation. Notably, both the p38 MAPK inhibitor SB203580 and TGP treatment downregulated NF-κB p65 expression in HaCaT cells, suggesting that TGP may ameliorate psoriasis through modulation of the p38 MAPK/NF-κB p65 signaling pathway [103].

##### 4.1.5.2 TGP ameliorates liver injury through MAPK/NF-κB pathway modulation

Drug-induced liver injury, characterized by medication-related hepatic damage, presents a substantial barrier during late-stage pharmaceutical research. Therapeutic administration of TGP significantly ameliorated methotrexate-induced hepatotoxicity, as evidenced by reduced liver index, improved histopathological alterations, and decreased serum ALT levels. Molecular analyses revealed that TGP treatment suppressed methotrexate-triggered phosphorylation of key signaling mediators, including p-Akt, p38-MAPK, and NF-κB p65 in hepatic tissues (*P* < 0.05) [41].

#### 4.1.6 TGP ameliorates autoimmune liver disease by the mitochondrial apoptotic pathway

TGP significantly reduced serum levels of ALT (*P* < 0.01), AST (*P* < 0.01), total bilirubin (*P* < 0.01), and γ-glutamyl transpeptidase (*P* < 0.05) in patients with autoimmune liver disease. Using concanavalin A (Con A)-induced autoimmune hepatitis (AIH) models, TGP pretreatment demonstrated protective effects by mitigating three key Con A-induced hepatocyte alterations: mitochondrial membrane potential collapse, ROS overproduction, and enhanced apoptotic activity. The levels of Bcl-2-associated X protein (Bax), cleaved caspase-3, and cytoplasmic cytochrome C decreased, while Bcl-2 and mitochondrial cytochrome C increased, suggesting TGP reduces hepatocyte apoptosis via the mitochondrial pathway. Additionally, TGP treatment inhibited bone marrow dendritic cells (DCs) maturation. Collectively, TGP ameliorates AIH by modulating hepatocyte apoptosis and DC maturation, demonstrating therapeutic potential for AIH treatment [42].

#### 4.1.7 TGP exhibits neuroprotective effects via cyclic adenosine monophosphate (cAMP)/protein kinase A (PKA)/cAMP response element-binding protein (CREB) signaling pathway

cAMP, the inaugural second messenger, controls cell function through multi-substrate kinase stimulation. It primarily acts through PKA and downstream effectors. The CREB, a ubiquitous transcription factor, critically regulates neuronal growth. Growth factor-mediated cAMP/PKA/CREB signaling enhances synaptic function, neuroprotection, and neurogenesis [104]. In neuropharmacology, TGP exhibits neuroprotective effects against 1-methyl-4-phenyl-1,2,3,6-tetrahydropyridine (MPTP)-induced experimental Parkinson's disease by inhibiting cell death and upregulating Bcl-2 expression (*P* < 0.05) through activation of the cAMP/PKA/CREB signaling pathway [43] (Fig. 4).

#### 4.1.8 TGP inhibits autoimmune responses by reducing the expression of programmed death-1 (PD-1)

The PD-1 protein and its ligand PD-L1 are inhibitory costimulatory molecules in the B7/CD28 superfamily, whose interaction forms an immunoregulatory axis that prevents hyperimmunity. Primary Sjögren's syndrome, an autoimmune disorder causing exocrine gland dysfunction, may involve lymphocyte impairment through soluble PD-1 (sPD-1)-mediated dysregulation of the PD-1/PD-L1 pathway in its pathogenesis. TGP demonstrates immunomodulatory effects by regulating the regulatory T (Treg)/Th17 cell balance: it suppresses autoimmune responses through the downregulation of PD-1 and sPD-1 expression, upregulation of the anti-inflammatory cytokine IL-10, and reduction of the pro-inflammatory IL-17 production [105].

#### 4.1.9 TGP exerts anti-inflammatory effects in bowel disease by targeting the p-Lyn/Snail regulatory axis

Through Lyn-mediated promotion of Snail nuclear translocation and stabilization by inhibiting ubiquitin-proteasome degradation, the transcription factor Snail negatively regulates epithelial junction proteins (E-cadherin and occludin), showing an inverse correlation with E-cadherin expression [106]. TGP exhibited a tremendous therapeutic effect on inflammatory bowel disease, an autoimmune disease closely related to the Lyn/Snail signaling pathway. Cao et al. [44] demonstrated that TGP dose-dependently reduces TNF-α, IL-17A, IL-23, and IFN-γ levels in both colon tissues and serum (*P* < 0.01 or *P* < 0.001). It also inhibits the production of p-Lyn and Snail, as well as Snail nuclear localization, favoring tight and adherent connections.

#### 4.1.10 TGP reduces neuroinflammation and depression by targeting NLRP3 inflammasome

Major depressive disorder is a critical public health concern, with a considerable impact on individuals' lives and the global burden of disease [50]. Elevated inflammatory markers are associated with both neural alterations and depression-like symptoms. TGP exerted a mitigating effect on depression-like behaviors in mice. This was accomplished through inhibiting the apoptosis of hippocampal neurons and decreasing the secretion of inflammatory cytokines. Mechanistically, TGP downregulated NLRP3 inflammasome components, including NLRP3, pro-caspase-1, caspase-1, IL-1β, and pyroptosis-related GSDMD-N, while attenuating mitochondrial dysfunction through enhanced mitophagy, thereby decreasing ROS accumulation and NLRP3 activation [45].

### 4.2 PF

As the primary active component of TGP, PF exhibits efficacy comparable to that of TGP, including anti-inflammatory and immunomodulatory effects. In addition to common small-molecule acids (e.g., BA), PF represents the most extensively studied component in RPA. Evidence indicates that PF exhibits significant therapeutic potential for various conditions, including neuronal damage, autoimmune diseases, and cancer treatment.

#### 4.2.1 The effect of PF on restoring nervous damage

Nervous system disorders impact either the central or peripheral nervous systems, marked by impairments in sensory, cognitive, and motor functions. These disorders include Parkinson's disease, Alzheimer's disease, stroke, amyotrophic lateral sclerosis, Huntington's disease, and cerebellar atrophy, among others.

##### 4.2.1.1 PF alleviating depression by regulating hypoxia-inducible factor-1 subunit alpha (HIF-1α)/miR-210/caspase-1/GSDMD signaling pathway

The GSDM family is characterized by a C-terminal repressor domain, a cytotoxic N-terminal domain, and a flexible linker region (except for GSDMF, an atypical family member). Inflammatory caspases (caspase-1, -4, -5, and -11) can cleave GSDM proteins, leading to the release of the N-terminal domain. This domain then assembles into large oligomeric pores in cell membranes, triggering pyroptosis. The process releases soluble intracellular components, including IL-1β and IL-18, through membrane rupture in swollen phagocytes [107].

**Fig. 6:**
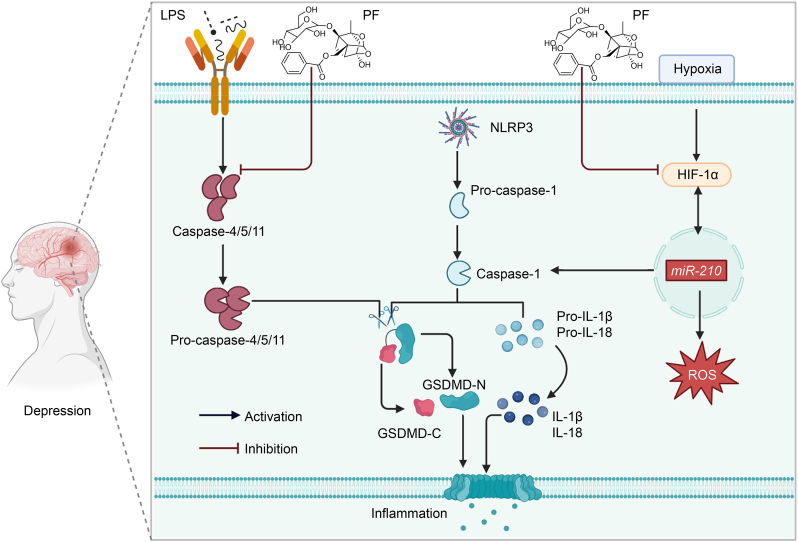
Paeoniflorin (PF) inhibits the hypoxia-inducible factor-1 subunit alpha (HIF-1α)/microRNA-210 (*miR-210*)/caspase-1/gasdermin (GSDM) D (GSDMD) signaling pathway, and reduces the release of inflammatory substances and the production of reactive oxygen species (ROS), thereby exerting therapeutic effects in depression. LPS: lipopolysaccharide; NLRP3: NLR family pyrin domain containing 3; GSDMD-C: GSDM D C-terminal domain; GSDMD-N: GSDM D N-terminal domain; IL-1β: interleukin**-**1β.

PF can improve hypoxia-induced brain infusion and achieve an anti-inflammatory effect on astrocyte pyroptosis under anoxia through HIF-1α/*miR-210*/caspase-1/GSDMD signaling pathway [46]. Moreover, other findings have suggested that PF exerts anti-depression effects by preventing the expression of caspase-11/GSDMD and other proteins implicated in pyroptosis signaling transduction, such as caspase-11, caspase-1, NLRP3, and IL-1β in the hippocampus of reserpine-treated mice [108] (Fig. 6).

##### 4.2.1.2 PF alleviating depression by regulating fibroblast growth factor-2 (FGF-2)/FGF receptor 1 (FGFR1) signaling pathway

**Fig. 7:**
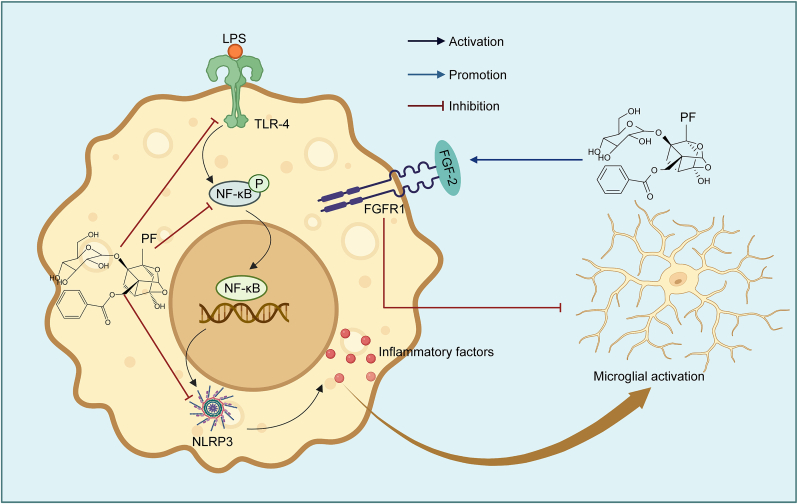
Paeoniflorin (PF) activates the fibroblast growth factor-2 (FGF-2)/FGF receptor 1 (FGFR1) signaling pathway, suppresses nuclear factor-kappa B (NF-κB) signaling transduction, and attenuates microglial activation in the hippocampus, thereby ameliorating depressive symptoms. LPS: lipopolysaccharide; TLR-4: Toll-like receptor 4; NLRP3: NLR family pyrin domain containing 3.

FGF-2 governs brain development by coordinating cell proliferation, migration, and angiogenesis, with FGFR1 being the primary mediator in adult hippocampal neurogenesis. PF exerts pronounced antidepressant and neuroprotective effects by inhibiting hippocampal microglial activation while activating the FGF-2/FGFR1 signaling pathway, and additionally attenuates frontal cortical FGF-2 overexpression in major depression [47] (Fig. 7).

##### 4.2.1.3 PF attenuates Parkinson's disease progression through JNK/p53 pathway regulation

The JNK signaling pathway is pivotal in regulating neuronal excitation, cell apoptosis, axonal growth, and memory formation. The tumor protein p53, essential for neuronal development and axonal regeneration, interacts with JNK, as this kinase is a critical mediator of p53-dependent apoptosis [109]. He et al. [48] discovered that PF inhibits the phosphorylation of the JNK/p53 signaling pathway, prevents damage to cornu ammonis 1 (CA1) and CA3 hippocampal neurons, and lowers hippocampus levels of p-c-Jun/Jun, p-JNK/JNK, and p-p53/p53, alleviating cognitive deficits in Parkinson's disease.

#### 4.2.2 PF regulates a variety of signaling pathways in the management of autoimmune disorders

Autoimmune diseases result from disrupted immune tolerance, where genetic and environmental factors trigger abnormal immune responses against self-antigens, causing tissue damage in conditions like rheumatoid arthritis, lupus, and ankylosing spondylitis [110]. Autoimmune disease pathogenesis remains unclear, and no targeted therapies currently exist for clinical use. Autoimmune disease has become a problem that cannot be ignored.

##### 4.2.2.1 PF alleviates autoimmune disorders by targeting NF-κB/IκBα and MAPK signaling

Like TGP, PF can regulate multiple NF-κB/IκBα and MAPK signaling pathways in different diseases. In allergic contact dermatitis, PF suppresses IFN-γ secretion and T-lymphocyte activation through the NF-κB/IκBα and p38 MAPK signaling pathways [111].

PF demonstrates therapeutic efficacy in autoimmune diseases, including LPS-induced macrophage activation, multiple sclerosis, and ovalbumin (OVA)-induced allergic asthma, through inhibition of NF-κB/IκBα and MAPK signaling pathways [49]. By preventing the activation of the p38 MAPK pathway, PF can reduce the expression of IL-22, an inflammatory cytokine crucial for epidermal cell growth and division, to treat psoriasis [50]. PF improved experimental autoimmune encephalomyelitis by inhibiting costimulatory molecules and IL-6 production of DCs (*P* < 0.05) *in vivo* and *in vitro*. The observed reduction in Th17 differentiation may result from concurrent inhibition of both IκB kinase (IKK)/NF-κB and JNK pathways [51].

Moreover, PF alleviates inflammatory reactions in ulcerative diseases via the NF-κB/IκBα pathway. PF can prevent the formation of NLRP3/Caspase-1 inflammasomes and the activation of NF-κB by inhibiting C−X−C motif chemokine receptor 2 (CXCR2) in diabetic foot ulcers [52]. PF demonstrates therapeutic efficacy in dextran sodium sulfate (DSS)-induced murine colitis and ulcerative colitis by suppressing NF-κB signaling, which downregulates pro-inflammatory mediators and reduces eosinophil infiltration [53].

##### 4.2.2.2 PF exerts therapeutic effects against autoimmune diseases through modulation of the PI3K/Akt signaling pathway

**Fig. 8:**
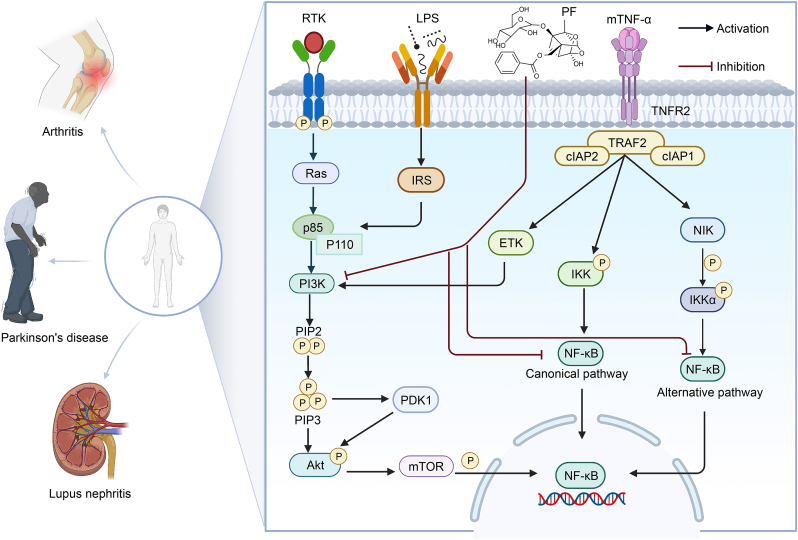
Paeoniflorin (PF) exerts therapeutic effects on autoimmune diseases by inhibiting the nuclear factor-kappa B (NF-κB) signaling pathway and phosphatidylinositol 3-kinase (PI3K)/protein kinase B (Akt) signaling pathway. RTK: receptor tyrosine kinase; LPS: lipopolysaccharide; mTNF-α: membrane-bound tumor necrosis factor alpha (TNF-α); Ras: rat sarcoma viral oncogene homolog; IRS: insulin receptor substrates; PIP2: phosphatidylinositol 4,5-bisphosphate; PDK1: 3-phosphoinositide-dependent protein kinase 1; mTOR: mechanistic target of rapamycin; TNFR2: tumor necrosis factor receptor 2; TRAF2: TNF receptor associated factor; cIAP2: cellular inhibitor of apoptosis protein 2; ETK: epithelial tyrosine kinase; IKK: inhibitor of NF-κB (IκB) kinase; NIK: NF-κB-inducing kinase.

The involvement of the PI3K/Akt signaling pathway and its downstream transcription factors in controlling cell growth, migration, proliferation, and metabolism in mammalian cells has been extensively studied. In response to extracellular stimuli, receptor tyrosine kinases are activated and subsequently recruit adaptor proteins, which bind and activate the regulatory PI3K regulatory subunit alpha (p85) subunit of PI3K for full PI3K activation. Activated PI3K enhances the phosphorylation of phosphatidylinositol 4,5-bisphosphate (PIP2) to phosphatidylinositol 3,4,5-trisphosphate (PIP3). PIP3 recruits 3-phosphoinositide-dependent kinase 1 (PDK1) and Akt membrane recruitment, initiating Akt phosphorylation by PDK1 [112]. Previously published results indicate that PF controls the PI3K/Akt pathways to decrease inflammation in animal models of autoimmune disorders, for instance mesangial proliferative glomerulonephritis, experimental arthritis, alpha-naphthylisothiocyanate-induced cholestasis, and so on [54,55] (Fig. 8).

##### 4.2.2.3 PF treats lupus nephritis via the mTNF-α-TNFR2 signaling pathway

The pleiotropic cytokine TNF-α exists in both soluble (sTNF-α) and transmembrane (mTNF-α) forms, mediating diverse immunological processes. Emerging evidence demonstrates that sTNF-α signaling via TNF receptor 1 (TNFR1) mainly promotes inflammation, while mTNF-α binding to TNFR2 mediates immune regulation and tissue regeneration. TNFR2 signaling is critical in autoimmune diseases [113]. PF strengthens the control of the mTNF-α-TNFR2 pathway, thus increasing CD4^+^Foxp3^+^ Treg cells. It can increase the expression level of mTNF-α in CD206^+^ macrophages and TNFR2 on CD4^+^Foxp3^+^ Treg cells, promote Treg cell expansion induced by macrophage M2 *in vitro*, thereby contributing to the treatment of lupus nephritis [56] (Fig. 8).

#### 4.2.3 The effect of PF on improving the safety and ingravidation of pregnant women during pregnancy

The gestational period refers to the physiological stage from conception to delivery, encompassing fetal development and preparation for parturition. During pregnancy, the mother's metabolism, digestive system, vascular system, nervous system, reproductive system, skeletal system and joint ligaments, and breasts undergo corresponding changes. Several medical conditions often accompany pregnancy, such as gestational diabetes, gestational hypertension, and gestational heart disease. These conditions can harm both the health of the mother and the growth and development of the fetus [114].

##### 4.2.3.1 PF alleviates preeclampsia by upregulating the expression of VEGF

Preeclampsia is a systemic vascular condition. Placental factors associated with widespread maternal endothelial dysfunction are likely to play a significant role in the pathophysiology of preeclampsia. Soluble fms-like tyrosine kinase-1 (sFlt-1), a soluble form of VEGF receptor, can inhibit VEGF signaling. Inhibiting VEGF and placental growth factors causes widespread endothelial dysfunction in preeclampsia [115]. Zhang et al. [57] investigated the preventive effects of PF on preeclampsia-related endothelial dysfunction. Their research showed that sFlt-1 and soluble endoglin (sEng), either alone or in combination, significantly inhibited human umbilical vein endothelial cells (HUVECs) migration, invasion, and tube formation while promoting apoptosis and suppressing VEGFA expression-effects that were substantially attenuated by PF pretreatment. Mechanistically, PF counteracted sFlt-1/sEng-mediated endothelial dysfunction through VEGFA upregulation.

##### 4.2.3.2 PF improves endometrial receptivity via leukemia inhibitory factor upregulation

Enhanced endometrial receptivity is pivotal for successful assisted reproductive technology outcomes via improved embryo implantation. PF improves the embryo implantation rate in the mouse embryo implantation failure model induced by mifepristone (RU486), enhances endometrial receptivity, and promotes adhesion between human trophectoderm-derived JAr cells and endometrial Ishikawa cells by upregulating leukemia inhibitory factor production *in vitro* [58].

#### 4.2.4 PF exerts anti-cancer effects through multiple molecular mechanisms

PF has been reported to exert an antitumor effect in nasopharyngeal carcinoma by downregulating neuronally expressed neural precursor cell expressed developmentally downregulated protein 4 expression and PTEN, and inhibiting p-Akt [59]. In addition, PF inhibits the growth of glioma cells by upregulating *miR-16* and downregulating MMP-9 expression [116]. PF alleviated cancer cachexia in colon 26 (C26) tumor-bearing mice, mitigating weight loss, muscle fiber atrophy, and functional decline. Mechanistically, PF concurrently suppressed TLR4/NF-κB signaling and activated Akt/mammalian target of rapamycin (mTOR) pathways in a C2C12 myotube atrophy cell model and C26 tumor-bearing cancer cachexia mice model, suggesting a dual-target approach against muscle wasting [60]. Moreover, PF demonstrates significant antitumor effects in gastric cancer cells (MGC-803 cell line) by concurrently suppressing cellular proliferation and promoting apoptosis through dual mechanisms involving *miR-124* upregulation and PI3K/Akt signaling pathway inhibition [61]. These findings highlight the considerable therapeutic value of PF in treating cancer.

#### 4.2.5 PF exerts anti-arthritis effects by inhibiting Th1 and Th17 cells in intestinal associated lymphoid tissues

PF treatment significantly suppressed Th1 (22.2%–23.1% reduction) and Th17 (25.4%–43.2% reduction) lineage-specific transcription factors in both mesenteric lymph nodes and Peyer's patches (*P* < 0.05), demonstrating that its anti-arthritic effects are mediated primarily through modulation of Th1/Th17 responses in gut-associated lymphoid tissues [62].

Clinical trial evidence has substantiated that PF exhibits considerable therapeutic potential for arthritis management. Data indicate that PF was comparable to conventional disease-modifying antirheumatic drugs (DMARDs) in improving rheumatoid arthritis symptoms, albeit with a modest effect size (20%). Notably, PF combined with cervus and cucumis polypeptide injection (CCPI) showed significantly fewer adverse events than DMARD regimens (*P* < 0.0001–0.05). These findings suggest that PF may serve as a safer alternative to DMARDs for long-term rheumatoid arthritis treatment, particularly when drug toxicity is a concern [117].

#### 4.2.6 PF ameliorates liver injury through multiple mechanisms

Liver injury encompasses structural and functional hepatic abnormalities arising from diverse etiologies, including short-term exposure to hepatotoxic agents (such as drugs and viruses) and chronic damage progressing to fibrosis or cirrhosis. PF alleviated LPS-induced acute liver injury in mice by enhancing mitochondrial function through multiple mechanisms, including increased SOD activity, reduced ROS and MDA levels, improved adenosine triphosphate (ATP) production, and enhanced activity of mitochondrial complexes I and III, while also upregulating key antioxidant proteins (SOD2 and forkhead box protein O1a (Foxo1a)) [118]. In the acetaminophen-induced liver injury model, PF reduced the levels of mitochondrial metabolic enzymes and suppressed the cytochrome P450 family 2 subfamily E member 1 (CYP2E1)/JNK signaling pathway, thereby mitigating acetaminophen-induced hepatotoxicity [63]. Additionally, PF ameliorated liver damage in hypercholesterolemic rats by modulating the Rho-associated coiled-coil-containing protein kinase (ROCK2)/AMP-activated protein kinase (AMPK)/nuclear sterol regulatory element-binding protein-1C (SREBP1C) signaling pathway [64]. In conclusion, PF has excellent potential in liver injury diseases.

#### 4.2.7 PF attenuates lung injury via immune-inflammatory modulation

In an OVA-induced murine asthma model, PF effectively attenuated lung injury by modulating the immune and inflammatory responses. Specifically, PF suppresses the generation of pro-inflammatory cytokines, reduces Th2 cell proliferation, and alleviates oxidative stress by restoring mitochondrial membrane potential, with these effects exhibiting dose dependency. Additionally, PF may regulate autophagy by modulating mitochondrial activity [119].

#### 4.2.8 PF alleviates coronary artery disease through the Wnt/β-catenin signaling pathway

Coronary artery disease is marked by dysregulated lipid metabolism, which results in lipid accumulation and the development of atherosclerotic plaques. The research indicated that in human coronary artery endothelial cells treated with oxidized low-density lipoprotein, PF notably boosted cell viability by 115.76%, inhibited apoptosis by 46.28%, and regulated the levels of inflammatory cytokines; specifically, it decreased IL-6 by 63.43% and IL-8 by 66.70% while increasing IL-10 by 181.15%. Moreover, PF administration effectively deactivated the Wnt/β-catenin signaling pathway, relieved aortic and aortic root plaque lesions, and improved serum lipid levels in high-fat diet-fed *ApoE*^−/−^ mice [65].

Clinical studies demonstrated that Xiongshao capsule, composed of chuangxiongol and PF, significantly reduced the incidence of recurrent angina at three-month (4.11%) and six-month (12.33%) follow-ups after percutaneous coronary intervention (PCI), while effectively inhibiting post-PCI lumen restenosis [120]. These findings highlight PF's therapeutic potential in cardiovascular disease management.

### 4.3 AF

AF is another major active component of TGP, sharing the same molecular formula as PF. Its pharmacological effects primarily include anti-inflammatory activity, tissue damage alleviation, and metabolic disease regulation. This section briefly summarizes recent advances in understanding AF and its pharmacological mechanisms.

#### 4.3.1 AF mitigates neuropathic pain through suppression of the MAPK/NF-κB signaling pathway

AF and PF exhibit comparable anti-inflammatory activities. In the context of neuroinflammatory disorders, AF and PF ameliorate pathological damage in chronic constriction injury rat models by inhibiting p38 MAPK pathway activation, modulating the expression of IL-1β and TNF-α, and suppressing p-JNK overexpression, thereby ultimately alleviating neuropathic pain [66].

#### 4.3.2 AF ameliorates depression by modulating the expression of both serotonin (5-HT) and brain-derived neurotrophic factor (BDNF)

Research has established that the dysregulation of critical neurotransmitter systems, particularly 5-HT, norepinephrine (NE), and dopamine (DA), constitutes a fundamental pathophysiological mechanism underlying depressive disorders. BDNF, a critical neurotrophic factor, plays a pivotal role in depression treatment through its elevated expression, which promotes neuronal growth and plasticity [121].

In chronic stress treatment experiments, Zhu et al. [67] demonstrated that AF modulates the nitric oxide (NO)-mediated signaling pathway in the hippocampus. This regulation elevates hippocampal levels of 5-HT, 5-hydroxyindoleacetic acid (5-HIAA), NE, and DA, while simultaneously increasing both protein expression in this pathway and BDNF levels. These changes ultimately ameliorated depression-like symptoms in rats, potentially through the neuroprotective effects mediated by 5-hydroxytryptamine 2A (5-HT2A) receptors. Similar to fluoxetine's pharmacological mechanism, AF appears to combat depression by modulating central nervous system neurotransmitters and elevating BDNF levels. Additionally, AF has shown efficacy in alleviating post-traumatic stress disorder-like symptoms and behavioral deficits in single-prolonged stress rats, potentially through stimulation of allopregnanolone production in the brain [68].

#### 4.3.3 AF ameliorates Alzheimer's disease by improving mitochondrial dysfunction

Alzheimer's disease is a kind of neurodegenerative disease characterized by the progressive deterioration of the nervous system, mainly manifested as aphasia, behavioral changes, and other aspects of dementia. Mitochondrial impairment and ultrastructural alterations, characterized by dysregulated expression of mitochondrial dynamics proteins such as increased dynamin-related protein 1 (DRP1) and decreased mitofusin 1 (MFN1), MFN2, and optic atrophy 1 (OPA1), represent early pathological manifestations in Alzheimer's disease progression, ultimately prompting mitochondrial dysfunction and cellular apoptosis [122]. AF improved mitochondrial dynamics in APP/PS1 mice by reducing DRP1 levels while increasing MFN1, MFN2, and OPA1 levels, and attenuated mitochondrial apoptosis via regulating Bcl-2 family proteins and suppressing caspase-3/cytochrome C activation in both hippocampus and cortex, along with reducing apoptotic cells in the anterior parietal cortex, thereby ameliorating Alzheimer's disease [69].

#### 4.3.4 AF alleviates ulcerative colitis by modulating the AMPK and NF-κB pathway

In a mouse model of DSS-colitis, AF treatment exerted protective effects via AMPK-mediated caudal-type homeobox 2 (CDX2) regulation and inflammatory cytokine modulation (IFN-γ, TNF-α, and IL-6), leading to improved mucosal integrity and reduced ulcerative lesions [70]. Additionally, in the same model, AF markedly decreased myeloperoxidase activity while upregulating forkhead box protein 3 (FOXP3) and STAT5 expression. It alleviated intestinal inflammation through inhibition of NF-κB activation and reduced phosphorylation of IκBα and p65 [123].

#### 4.3.5 AF plays a vital role in metabolic disease by regulating the AMPK and PI3K/Akt pathways

AF demonstrates anti-obesity effects by modulating both the AMPK and PI3K/Akt signaling pathways. This therapeutic activity was observed in both human adipose-derived MSCs and high-fat diet-induced obese mice [71]. In the type 2 diabetes model induced by a high-fat diet and a low dose of streptozotocin, AF reduces blood glucose concentration and improves glucose metabolism under insulin stimulation. It has beneficial effects on preventing non-alcoholic steatohepatitis, as evidenced by decreased AST levels in plasma and reduced TNF-α mRNA expression in the liver [72].

### 4.4 BPF

BPF is a monoterpene compound with a structure formed by the addition of a benzoyl group at the 4′-OH position of PF. Research on the pharmacological effects of BPF has demonstrated that it exerts anti-allergic effects and affects respiratory diseases, cardiovascular diseases, and other related conditions.

#### 4.4.1 BPF prevents allergies by blocking the MAPK signal pathway

Allergic diseases are typical immune-related disorders, such as pollen allergy, food allergy, and allergic asthma, that result from immune system dysfunction, with current treatments primarily targeting IgE-mediated mast cell activation. BPF alleviates allergic responses by suppressing the MAPK signaling pathway (ERK1/2, JNK, and p38), consequently reducing pro-inflammatory cytokines (IL-3, IL-4, IL-5, and IL-13; *P* < 0.05) and protecting cellular structural integrity at low concentrations [73].

#### 4.4.2 BPF attenuates sepsis progression through inhibition of NF-κB and MAPK phosphorylation

Sepsis is a condition with potentially fatal organ failure induced by an unregulated host response, and it may represent the immune system's response to injury. In the LPS-induced model, BPF attenuated the activation of NF-κB and MAPK (p38/JNK/ERK) activation in human HUVECs and THP-1 cells, subsequently reducing levels of inducible NO synthase (iNOS), TNF-α, and IL-6 (*P* < 0.05). Furthermore, BPF decreased TNF-α and C−X−C motif ligand 1 (CXCL1) (*P* < 0.05) as well as other inflammatory factors, ultimately improving survival rates in mice exposed to lethal LPS doses. These results demonstrate BPF's therapeutic potential for sepsis treatment [74].

#### 4.4.3 BPF attenuates hepatic lipid accumulation through AMPK activation

Fatty liver disease is a pathological condition characterized by abnormal accumulation of fat (primarily triglycerides) within liver cells. Organic cation transporter 1 (OCT1) is exclusively expressed in the liver and mediates hepatocyte uptake of cationic therapeutics and endogenous compounds. It is highly expressed in normal human hepatocytes, functioning as a drug transporter; thus, inhibiting OCT1 could be a viable therapy for fatty liver disease [124]. In both OCT1-overexpressing L02 (normal hepatocyte) and HepG2 (hepatoma) cell lines, BPF treatment significantly attenuated free fatty acid-induced lipid accumulation (*P* < 0.05). Mechanistically, it activated AMPK while downregulating lipogenic enzymes, fatty acid synthase, and acetyl-CoA carboxylase, thereby preventing hepatocyte steatosis. These dual effects on lipid metabolism underscore BPF's therapeutic potential for fatty liver diseases [75].

### 4.5 OPF

The structure of OPF has an additional hydroxyl group on the benzene ring of PF. Therefore, the polarity of OPF is greater than that of PF. OPF has a series of biological activities, including improving myocardial ischemia, protecting against acute lung injury, and treating diabetic nephropathy.

#### 4.5.1 OPF improves myocardial I/R (MI/R) injury via silent information regulator sirtuin 1 (SIRT1)/Foxo1 signaling

MI/R injury refers to the phenomenon where, after complete or partial coronary artery occlusion, reperfusion within a specific time window causes additional damage to the myocardial tissue despite successful restoration of blood flow [125]. SIRT1, a pivotal member of the sirtuin family, plays an increasingly recognized crucial role in MI/R injury by exerting cardioprotective effects through Foxo1 deacetylation-mediated suppression of apoptotic pathways, thereby attenuating cellular damage. OPF significantly mitigates cardiac damage and improves cardiac function by reducing myocardial infarction-related cardiac injury markers (creatine kinase and cardiac troponin I/T) while enhancing H9c2 cardiomyocyte viability. These cardioprotective effects are mediated through the SIRT1/Foxo1 signaling pathway, which inhibits apoptosis, ultimately alleviating MI/R [76].

#### 4.5.2 OPF protects acute lung injury by regulating the TLR and PTEN/Akt signaling pathway

Acute lung injury involves pulmonary edema, alveolar barrier damage, oxidative stress, and inflammation. In LPS-induced RAW264.7 macrophages, OPF significantly suppressed NO production and downregulated iNOS expression. Mechanistically, OPF attenuated LPS-induced inflammation by modulating the TLR signaling pathway, while concurrently inhibiting the phosphorylation of ERK and p38 MAPK, thereby exerting anti-inflammatory effects [77]. Further supporting its therapeutic potential, a study by Fan et al. [78] demonstrated that OPF treatment alleviated LPS-induced pulmonary edema, inflammation, and oxidative stress in acute lung injury mice, improving survival rates. This protective effect was associated with enhanced SIRT1 expression and activity, which safeguarded alveolar epithelial cells via regulation of the PTEN/Akt pathway.

#### 4.5.3 OPF alleviates diabetic nephropathy through suppression of oxidative stress and inflammatory responses

Advanced glycation end products (AGEs) contribute to renal injury by promoting glomerular basement membrane thickening and kidney tissue damage. Oxidative stress and inflammation-related reactions induced by AGEs play a crucial role in diabetic nephropathy and mesangial cell injury. In the co-culture system of macrophages and mesangial cells (HBZY-1), the migration of macrophages was inhibited by OPF. OPF significantly reduced the production of AGE-induced inflammatory factors, including IL-6 (*P* < 0.05) and MCP-1 (*P* < 0.01) [79].

## 5 PGG

Previously, *in vitro* and *in vivo* experiments have shown that PGG has many pharmacological and biological activities, such as antimicrobial activity and preventing diabetic digestive diseases. PGG is a polyphenolic compound classified as a tannin that has demonstrated remarkable potential in anticancer and antioxidant activities in recent years. The unique chemical structure of PGG involves five ester bonds connecting the carboxyl group of gallic acid with the aliphatic hydroxyl groups of glucose. Notably, this structure enhances the anti-inflammatory and antioxidant activities through its hydroxyl groups [126]. Recent studies have revealed that PGG demonstrates biological activity against various cancer cells, highlighting its potential as an effective anticancer agent [127]. Notably, numerous studies have explored its efficacy in cancer treatment using both *in vitro* and *in vivo* models.

### 5.1 PGG defenses against invading microbiota

The microbiota is the population of microorganisms that reside within the host, including bacteria, viruses, fungi, and protozoans. When microbiota infect their host at mucosal or epithelial surfaces, they can cause severe discomfort and even death. PGG can defend against invading pathogens such as viruses and some harmful bacteria.

Hepatitis C virus (HCV) infects susceptible people mainly through blood transmission. PGG exerts its efficacy when the HCV attaches to cells. It causes minor damage to the integrity of HCV particles and does not stop the replication process of RNA. When co-administered with the clinically validated HCV inhibitor daclatasvir, PGG exhibits complementary antiviral effects [80].

The neurotropic rabies virus (RABV) gains entry to human hosts predominantly through severe animal-inflicted skin breaches. Mechanistically, RABV infection induces *miR-455-5p* to downregulate suppressor of cytokine signaling 3 (SOCS3) through its direct binding to the SOCS3 3′ untranslated region. This suppression results in STAT3 overactivation, which upregulates IL-6 expression and ultimately triggers neuroinflammation. Notably, PGG demonstrates anti-rabies activity by inhibiting *miR-455-5p* synthesis [81].

Prion diseases are degenerative neurological disorders triggered by the conformational conversion of normal cellular prion protein (α-helix-rich and β-sheet-deficient) into a β-sheet-rich pathological isoform. The treatment of PGG can inhibit the fibrosis of prion protein, reduce the production of prion protein fibers, significantly decompose prion protein fibrils, and maintain the α-helix structure. PGG may be a potential drug for treating prion diseases [128].

### 5.2 PGG ameliorates inflammatory bowel disease by inhibiting the MyD88/NF-κB pathway

**Fig. 9:**
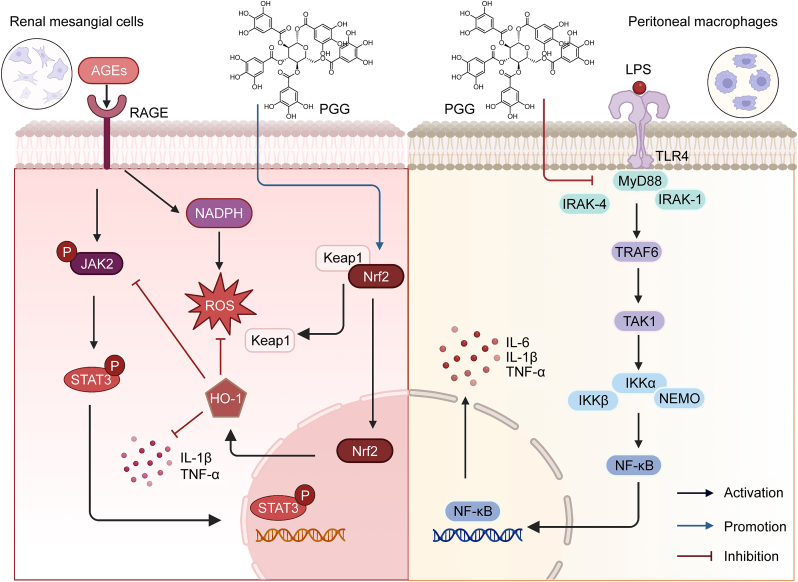
1,2,3,4,6-penta-*O*-galloyl-β-D-glucose (PGG) ameliorates diabetic nephropathy by inhibiting the Janus kinase 2 (JAK2)/signal transducer and activator of transcription 3 (STAT3) cascade through activation of nuclear factor erythroid 2-related factor 2 (Nrf2)/heme oxygenase-1 (HO-1) signaling. PGG ameliorates inflammatory bowel disease by inhibiting the myeloid differentiation factor-88 (MyD88)/nuclear factor-kappa B (NF-κB) pathway. AGEs: advanced glycation end-products; RAGE: receptor for advanced glycation end-products; NADPH: nicotinamide adenine dinucleotide phosphate; ROS: reactive oxygen species; Keap1: Kelch-like ECH-associated protein 1; IL-1β: interleukin**-**1β; TNF-α: tumor necrosis factor alpha; LPS: lipopolysaccharide; TLR4: Toll-like receptor 4; IRAK-4: IL-1 receptor-associated kinase 4; TRAF6: TNF receptor-associated factor 6; TAK1: transforming growth factor-beta (TGF**-**β)-activated kinase 1; IKKα: inhibitor of NF-κB (IκB) kinase subunit alpha; NEMO: NF-κB essential modulator.

TLR4/myeloid differentiation factor 88 (MyD88) is a typical inflammatory pathway, and research has demonstrated that TLR4 expression is significantly up-regulated in mice with colitis. Through IL-1R-associated kinase (IRAK), TIR domain containing adaptor protein (TIRAP) recruits MyD88 to TLR4, a process that induces the phosphorylation of downstream IRAK1, transforming growth factor beta (TGF**-**β)-activated kinase 1 (TAK1), and IKKβ. This, in turn, activates NF-κB, which promotes the secretion of pro-inflammatory cytokines such as IL-1β and TNF-α, ultimately leading to inflammation [129]. Jang et al. [82] demonstrated that in LPS-stimulated peritoneal macrophages, PGG suppressed NF-κB activation by directly binding to MyD88, thereby inhibiting TLR signaling and downregulating pro-inflammatory cytokines (TNF-α, IL-1β, and IL-6; *P* < 0.05). In 2,4,6-trinitrobenzene sulfonic acid (TNBS)-induced colitis mice, oral PGG administration attenuated colon shortening and myeloperoxidase activity while reducing NF-κB activation and pro-inflammatory cytokine levels (IL-1β, TNF-α, and IL-6; *P* < 0.05) (Fig. 9).

### 5.3 PGG alleviates peptic ulcers by neutralizing gastric H^+^/K^+^-ATPase activity

Peptic ulcers, chronic lesions occurring in the stomach and duodenum, result from gastric mucosal digestion by acidic gastric juice, a process that is critically dependent on gastric H^+^/K^+^-ATPase-mediated acid secretion in the final step. Ono et al. [130] found that PGG can effectively inhibit gastric H^+^/K^+^-ATPase, thereby reducing gastric acid secretion, and it has been used to treat peptic ulcers in Japan. In addition, PGG has been found to inhibit various enzymes, such as salivary α-amylase, angiotensin-converting enzyme, endopeptidase, and cyclooxygenase [131].

### 5.4 PGG attenuates diabetic nephropathy by activating the nuclear factor erythroid 2-related factor 2 (Nrf2)/heme oxygenase-1 (HO-1) pathway, thereby suppressing JAK2/STAT3 signaling

Diabetic nephropathy, a critical complication of diabetes mellitus, represents the primary cause of end-stage renal disease. Studies have shown that inflammation is a significant cause of this disease. The JAK/STAT pathway mediates inflammatory responses, while oxidative stress serves as a key trigger for diabetic nephropathy pathogenesis. The excessive production of ROS activates the JAK/STAT cascade, thereby promoting the development of inflammation. A key antioxidant enzyme, HO-1, catalyzes the conversion of heme to ferrous iron, carbon monoxide, and biliverdin. Nrf2 can initiate Nrf2-mediated transcription to activate the antioxidant enzyme HO-1 [132]. Nrf2/HO-1 is pivotal to removing ROS and maintaining cellular redox homeostasis. The results demonstrated that PGG significantly suppressed AGEs-induced ROS generation while reactivating the Nrf2/HO-1 antioxidant pathway, which had been inhibited by AGEs. While ML385 blocked Nrf2/HO-1 signaling and exacerbated oxidative/inflammatory responses (ROS and cytokines) and JAK2/STAT3 activation, PGG restored Nrf2/HO-1 function, effectively normalizing these pathological processes [83] (Fig. 9).

### 5.5 PGG attenuates lung injury through multiple protective mechanisms

Emphysema is characterized by chronic inflammation, oxidative stress, alveolar rupture, etc. It is a pathological state where the elasticity of the distal end of the terminal bronchus decreases, leading to an increase in lung capacity and lung inflation, which is accompanied by the destruction of the airway wall [84]. MMP-12 activity was considerably reduced in the lungs of emphysema animals treated with PGG, and PGG protected elastin while inhibiting elastase breakdown (*P* < 0.05) [133]. In addition, PGG can protect against acute lung injury induced by LPS to some extent, reduce the total number of cells and the count of polymorphonuclear leukocytes in bronchoalveolar lavage fluid (*P* < 0.01), reduce the Wet/Dry ratio (*P* < 0.05) and protein concentration (*P* < 0.01) of the lung, reduce lung permeability, and reduce the damage caused to the lung [85]. Apoptosis is a significant factor in acute lung injury. AMPK can activate the PI3K/Akt pathway to regulate cell death. Akt phosphorylation can increase Nrf2 nuclear translocation and cellular antioxidant activity. PGG mitigates acute lung injury via AMPK/PI3K/Akt/Nrf2 pathway activation [86].

### 5.6 PGG exerts anticancer effects through multiple pharmacological mechanisms

Despite therapeutic advances, cancer maintains its status as a leading cause of global morbidity and mortality. Accumulating evidence has proved that PGG has preventive and therapeutic effects on cancer. Cellular studies have tested PGG in cancer cell cultures. PGG reduces the growth of estrogen-sensitive human breast cancer MCF-7 cells and the phosphorylation and protein levels of estrogen receptor α by regulating the erythroblastic leukemia viral oncogene homolog (ERBB)/PI3K/Akt pathway and lysosomal consumption [87]. In another study, PGG inhibited the growth of SK-HEP-1 cells, an HCC cell line, by blocking the G1/G0 phase of the cell cycle and inhibiting the activation of NF-κB [88]. Additionally, Kawk et al. [89] found that PGG can induce the expression of p53 and reduce the proliferation of colon cancer cells. Studies have found that PGG inhibits many cancer cell lines from growing, including colorectal, cervical, leukemia, liver, head and neck, lung, breast, prostate, and pancreatic cancer cells [134]. The hydrophobic nature of PGG has constrained pharmacokinetic profiling and safety assessment, necessitating further investigation to validate its clinical applicability for cancer treatment and translation.

PGG is also potentially therapeutic in treating calcium chloride-induced abdominal aortic aneurysm (AAA) in rats. AAA is an abnormal dilatation of the aorta. If left untreated, the abnormally dilated abdominal aorta frequently ruptures, resulting in a much higher fatality risk. PGG can significantly reduce the aortic dilatation, maintain the aortic elastin structure, and prevent the further development of an aneurysm [135]. Data indicate that in AAA, most arterial lumens contain thrombi, which may affect the delivery of PGG to the aortic wall. Intravenous and intraperitoneal delivery routes of PGG as potential therapies for AAA are feasible in animal models [14]. The oral route of PGG delivery is challenging due to its limited bioavailability. In individuals with early AAA, PGG may be a potential approach to restore the aorta's structure and function.

## 6 The relationship between RPA's material basis and its pharmacological effects

RPA, one of the most extensively used TCMs in clinical practice, is frequently employed to treat various disorders, including liver diseases, rheumatoid arthritis, asthma [4], psychiatric disorders, and autoimmune diseases etc. [34]. The monoterpene glycosides and tannins in RPA contribute to its pharmacological effects. Therefore, we present an overview of the medicinal properties of RPA to clarify the correlation between these bioactive components and its therapeutic efficacy. Currently, many researchers focus on the pharmacological effects of RPA, spanning gastrointestinal disorders to cancers. Subsequently, we summarize the pharmacological effects and mechanisms of RPA.

### 6.1 RPA treats miscarriages and beneficial embryonic development

Due to its analgesic and antispasmodic properties, RPA was identified as one of the top six herbs most frequently used to prevent miscarriages in published clinical trials [136]. Miscarriages are primarily considered a result of failed embryonic development, and immunological dysregulation may play a key role in this process. Xu et al. [137] reported that the Th1/Th2/Th17 cytokine network is related to embryonic safety and suggested that RPA may support embryo growth by downregulating fetal Th1/Th2/Th17 cytokines and their receptors. In Section 4.2.3, literature analysis indicates that PF, a monoterpene glycoside, markedly increases embryo implantation rates by improving endometrial receptivity. These observations suggest that the therapeutic effects of RPA in preventing miscarriage may be attributable, at least in part, to its bioactive monoterpene glycosides.

### 6.2 RPA in the treatment of allergic asthma

Allergic asthma is a respiratory condition characterized by an IgE-mediated reaction to inhaled allergens. RPA has been widely used to treat asthma in TCM hospitals. Similar to its use in preventing miscarriages, RPA treats asthma through its antispasmodic effects. Our previous research has extensively studied the pharmacodynamic material basis of RPA, demonstrating that it possesses broad anti-allergic asthma effects [4]. We tested the bioactive components of RPA in OVA-induced asthmatic mice and identified the main compounds using ultra performance liquid chromatography (UPLC) compared with standards. PF, AF, PGG, and TGG were identified as the key components contributing to RPA's pharmacological activity [4]. Our parallel investigation demonstrated that both TGP and PF effectively suppressed OVA-induced asthmatic features in murine models. PF inhibited oxidative stress through dose-dependent recovery of mitochondrial membrane potential and metabolic activity regulation, while concurrently suppressing autophagy via mitochondrial function modulation [119].

Based on the above systematic analysis, RPA contains two major classes of bioactive compounds that have demonstrated therapeutic potential for asthma and pulmonary diseases: monoterpene glycosides (TGP, PF, and OPF) and tannins (e.g., PGG). Notably, the observed pulmonary benefits may be predominantly mediated by monoterpene glycosides, as evidenced by their multifaceted pharmacological activities in improving respiratory function.

### 6.3 RPA is utilized in the treatment of gastrointestinal disorders

The cause of ulcerative colitis remains unknown, but the condition is characterized by chronic mucosal ulceration in the rectum and colon. Multiple factors contribute to its pathogenesis, including genetics, environment, and dysregulated mucosal immunity. Yan et al. [138] demonstrated that RPA dispensing granules effectively alleviated DSS-induced colitis in mice, reducing the severity of weight loss, colon shortening, and pro-inflammatory cytokine levels. Comprehensive analyses in these studies demonstrated that RPA aqueous extract upregulates tight junction components (zonula occluden-1 (ZO-1), occludin, and claudin-1) while attenuating inflammatory responses through regulation of the IL-23/IL-17 axis. Additionally, RPA treatment increased the relative abundance of beneficial gut bacteria and improved microbial diversity. These findings indicate that both monoterpene glycosides (TGP, AF, and PF) and tannins (PGG) isolated from RPA show therapeutic potential against intestinal disorders. While monoterpene glycosides contribute significantly to RPA's intestinal protective properties, PGG shows particular promise in treating a spectrum of gastrointestinal pathologies, such as ulcerative colitis, inflammatory bowel disease, and colorectal cancer.

### 6.4 RPA significantly improves the manifestation of cancer

Cancer cachexia, a severe complication affecting cancer patients, manifests as progressive depletion of skeletal muscle and adipose tissue, ultimately resulting in compromised muscle function. Ubiquitin-dependent proteolysis serves as a central mechanism driving this muscle wasting process. It was found that an ethanol extract of RPA (hereafter referred to as RPA-EtOH) could significantly downregulate the expression levels of muscle-specific E3 ubiquitin ligases, muscle RING finger-1 (MuRF1), and muscle atrophy F-box (MAFbx) in skeletal muscle tissue of tumor-bearing mice and restore myosin heavy chain (MHC) levels in muscle. RPA-EtOH inhibits NF-κB signaling and decreases inflammatory cytokines, such as TNF-α, IL-6, and IL-1β [139].

Bladder cancer refers to malignant tumors in the bladder mucosa. Cancer progression is assumed to be linked to the dysfunction of cell cycle checkpoints. RPA can inhibit the proliferation of human bladder cancer TSGH-8301 cells, arresting them in the G2/M phase both *in vitro* and *in vivo*. In TSGH-8301 cells, RPA inhibits the expression of cyclin B1, cell division cycle 2, cell division cycle 25B, and p-checkpoint kinase 2 (p-Chk2). Furthermore, when administered to rats at doses of 0.5 and 1 g/kg, RPA significantly increased p-Chk2, suppressing bladder cancer progression [140]. In addition, RPA exhibits tonic properties with broad therapeutic and preventive applications, demonstrating significant efficacy in alleviating cancer-related pain and enhancing immune function to bolster patients' resistance against malignancies [141].

Based on the comprehensive analysis of the above two types of compounds, it was discovered that the active components of RPA (TGP, PF, and PGG) exhibit broad-spectrum anticancer activity through distinct yet complementary mechanisms. TGP inhibits breast cancer growth by inhibiting NF-κB/CCL2 signaling [39]. PF inhibits the growth of nasopharyngeal carcinoma and gastric cancer cells by blocking PI3K/Akt signaling [61]. PGG arrests the cell cycle at G1/G0 phase and suppresses NF-κB activation, halting proliferation across multiple cancer cell lines [88]. It is worth noting that existing evidence indicates limited oral bioavailability of PGG *in vivo*, while its antitumor effects have been primarily demonstrated *in vitro* studies. Although PGG exhibits potent anticancer potential, its efficacy via oral administration requires further validation. Collectively, these findings demonstrate that TGP, PF, and PGG modulate the tumor microenvironment to suppress cancer progression, revealing their essential contributions to RPA's anticancer efficacy through a multi-target mechanism that confers therapeutic advantages for cancer treatment.

### 6.5 RPA protects liver injury

Hepatic stellate cells (HSCs) are crucial in developing liver fibrosis. Platelet-derived growth factor BB (PDGF-BB) is widely regarded as the most potent mitogen and chemoattractant for HSCs. Studies have shown that RPA extracts alleviate liver injury by inhibiting PDGF-BB-induced HSC chemotaxis, as well as the expression of α-smooth muscle actin and collagen [142]. Additionally, saikosaponins inhibit the activity of hepatic glutathione synthetase (GSS), contributing to liver injury. However, RPA can increase the abundance of glycosidase-producing gut bacteria, enhancing the conversion of saikosaponins to saikogenins *in vivo*. This conversion reduces the inhibitory effect of saikosaponins on GSS activity and helps maintain hepatic redox balance [143]. Our previous studies demonstrated that a bioactive fraction of RPA, mainly containing monoterpene glycosides and tannins, mitigates bile duct ligation-induced hepatic fibrosis by inhibiting NLRP3-mediated pyroptosis [144].

Based on the above summary, we found that multiple monoterpene glycosides (TGP, PF, and BPF) exert hepatoprotective effects through pleiotropic mechanisms, including inhibition of NLRP3-mediated pyroptosis [144], suppression of the MAPK/NF-κB pathway, and scavenging of mitochondrial ROS [41]. These collective findings identify monoterpene glycosides as the principal mediators of RPA's hepatoprotective efficacy.

### 6.6 RPA inhibits calcium oxalate nephrolithiasis

Nephrolithiasis is a complex disease with a global prevalence. In the RPA extract treatment group, it significantly reduced urinary and renal oxalate levels while markedly increasing urinary calcium and citrate excretion. Additionally, crystal formation was strongly suppressed. Compared to the control group, the RPA-treated group showed a significant decrease in osteopontin expression and reduced inflammatory infiltration in renal tissue [145]. Collectively, these data support RPA as a potential prophylactic intervention against calcium oxalate nephrolithiasis through its dual modulation of urinary lithogenic factors.

Although RPA has been relatively understudied in renal diseases, accumulating evidence suggests its significant therapeutic potential. Based on the above summary, it was discovered that both monoterpene glycosides and PGG from RPA exhibit therapeutic effects against kidney diseases. Furthermore, traditional medicine recognizes RPA for its liver- and kidney-nourishing properties. The evidence of its effective components and traditional applications supports the potential of RPA in treating kidney diseases.

### 6.7 RPA is effective against depression

Radix Bupleuri-RPA drug pair administration significantly reduced immobility time in both the forced swim and tail suspension tests, while attenuating reserpine-induced hypothermia in mice. Furthermore, Radix Bupleuri-RPA drug pair pretreatment markedly elevated levels of epinephrine and 5-HT in hippocampal and cortical tissues [146]. In clinical practice, Radix Bupleuri and RPA form the fundamental herb pair for liver constraint relief and mood regulation [143], serving as the core components of multiple classical antidepressant formulas, including Sini powder, Chaihu Shugan powder, and Xiaoyao powder. These findings reveal that RPA demonstrates potent antidepressant-like effects by modulating monoaminergic systems and activating neuroprotective pathways.

Based on our systematic review of monoterpene glycosides' pharmacological activities and mechanisms, combined with recent literature evidence, the active components of RPA (TGP, PF, and AF) demonstrate significant antidepressant efficacy. The potential mechanisms may involve: elevating monoamine neurotransmitter levels [67], normalizing hypothalamic-pituitary-adrenal axis dysfunction, repairing neuronal damage, etc. [45]. These findings collectively indicate that monoterpene glycosides serve as the primary active constituents mediating RPA's antidepressant properties.

### 6.8 RPA has an anti-atopic dermatitis effect

Atopic dermatitis involves epidermal dysfunction driven by Th2-mediated immunity, barrier defects, and severe itching. Experimental studies demonstrated that topical application of 1% or 6% RPA root extract for three weeks significantly reduced scratching behavior in mice with 2,4-dinitrochlorobenzene (DNCB)-induced atopic dermatitis. Furthermore, the treatment decreased key atopic dermatitis biomarkers, including serum IgE levels, IgG1/IgG2a ratios, and splenocyte production of IL-4 and IFN-γ. These results reveal RPA extract mechanistically modulates the immunological abnormalities and clinical manifestations of atopic dermatitis [147].

RPA has been used in TCM for over 1000 years to treat pain, inflammation, and immune disorders. Our aforementioned summary indicates that these therapeutic effects can be attributed to its monoterpene glycosides, which exhibit broad anti-inflammatory and immunomodulatory effects through multiple mechanisms: PF inhibits inflammation in various autoimmune models including experimental arthritis [62], psoriasis [50], and experimental autoimmune encephalomyelitis [51]; TGP has demonstrated clinical benefits in managing autoimmune conditions such as rheumatoid arthritis [35], systemic lupus erythematosus [34], and psoriasis [36]. Additional monoterpene glycosides, including AF and BPF, show promising anti-allergic effects. In summary, these findings demonstrate that monoterpene glycosides likely serve as the primary active constituents mediating RPA's immunomodulatory effects.

## 7 Conclusion and future perspectives

**Fig. 10:**
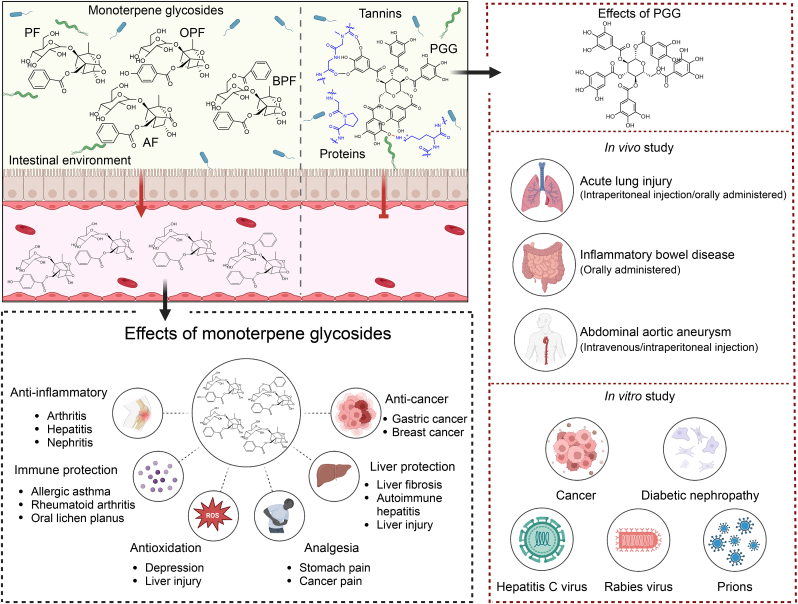
Monoterpene glycosides and tannins have been identified as the two primary natural bioactive compounds in Radix Paeoniae Alba (RPA). Monoterpene glycosides can be absorbed into the systemic circulation and exhibit various pharmacological properties including anti-inflammatory, immunoprotective, antioxidant, analgesic, hepatoprotective, and anticancer effects. 1,2,3,4,6-penta-*O*-galloyl-β-D-glucose (PGG) forms insoluble complexes with salivary proteins via hydrogen bonding and hydrophobic interactions, thereby reducing systemic absorption. PGG demonstrates anticancer, antiviral, and therapeutic efficacy against lung injury and inflammatory bowel disease. PF: paeoniflorin; AF: albiflorin; OPF: oxypaeoniflorin; BPF: benzoylpaeoniflorin.

As a commonly used TCM in clinical practice, RPA exhibits significant therapeutic effects in various liver diseases, rheumatoid arthritis, asthma, psychiatric disorders, and autoimmune diseases. Studies on the active constituents of RPA have identified monoterpene glycosides and tannins as two main natural bioactive compounds. However, the basic research on RPA has mainly focused on monoterpene glycosides. Both monoterpene glycosides and their gut metabolites demonstrate systemic bioavailability through intestinal absorption into the systemic circulation. Our systematic analysis reveals that these compounds exhibit predominant therapeutic effects across several key areas: immunomodulation, anti-inflammatory actions, hepatoprotection, and antidepressant activity. Notably, tannins from RPA, which contain highly hydrophobic polyphenols generate limited oral bioavailability. Compared to monoterpene glycosides, PGG demonstrates a more focused therapeutic profile, primarily exhibiting efficacy in three domains: antiviral applications, anticancer interventions, and gastrointestinal diseases (Fig. 10). Little research has been conducted on the decomposition products of tannins in blood. Although tannins have been studied for treating non-gastrointestinal diseases via the oral route, we speculate that they may act by regulating metabolism to display their function. These well-characterized pharmacological properties substantiate RPA's traditional uses in corresponding disease conditions, with the absorbed monoterpene glycosides and tannins serving as the material basis for its clinical efficacy.

This study provides a systematic investigation of RPA's therapeutic effects by exploring the pharmacological relationship between its tannins and monoterpene glycosides, analyzing the bioavailability profiles of these compound classes, and elucidating their mechanistic actions through diverse signaling pathways and molecular targets. However, several critical limitations must be acknowledged. Although monoterpene glycosides (particularly TGP and PF) have been widely applied clinically, most pharmacological data on AF, BPF, OPF, and PGG remain preclinical, relying predominantly on rodent models that may not fully recapitulate human pathophysiology. Although PGG and PF exhibit strong anticancer effects *in vitro* and in animal models by suppressing tumor cell proliferation via multiple mechanisms, their clinical effectiveness has not yet been confirmed in randomized controlled trials. Furthermore, pharmacokinetic analyses (e.g., TCMSP database) reveal concerning bioavailability limitations: except for PF, these compounds exhibit poor intestinal absorption and low blood bioavailability, raising questions about their translational potential. These limitations highlight the critical need for developing innovative delivery strategies, such as liposomal/nanoparticle systems or structural modifications, to enhance their therapeutic potential, particularly for localized applications in the gastrointestinal tract.

Additionally, the pharmacological efficacy and clinical applicability of drugs fundamentally depend on the elucidation of their mechanisms of action and therapeutic targets. As critical regulators of cellular processes, signaling pathways represent key intervention points for modern therapeutics. Our investigations demonstrate that RPA exerts its multifaceted therapeutic effects through two primary bioactive compound classes: monoterpene glycosides and tannins, which modulate multiple signaling pathways, including both canonical (PI3K/Akt, NF-κB/MAPK, JAK-STAT, NLRP3/caspase-1, and JNK/p53) and non-canonical pathways (Nrf2/HO-1 and SIRT1/Foxo1). Through these pathways, they regulate critical cellular processes such as proliferation and apoptosis in cancer, inflammatory responses in autoimmune diseases, and metabolic homeostasis in various disorders. This multi-target pharmacological profile enables RPA to simultaneously intervene in diverse disease processes more effectively than single-pathway inhibitors, particularly in complex multifactorial diseases.

It is worth noting that the potential synergies between monoterpene glycosides and tannins remain unexplored. Addressing these gaps through human-relevant models, clinical trials, bioavailability optimization, and interaction studies would significantly enhance the clinical translation of RPA and its active constituents.

## CRediT authorship contribution statement

**Qitong Zheng:** Writing – original draft, Investigation. **Mengyao Chen:** Writing – review & editing, Visualization. **Jialiang Ying:** Writing – review & editing, Visualization. **Zhichao Wang:** Writing – review & editing, Supervision. **Qiyuan Shan:** Writing – review & editing, Supervision. **Xia-Nan Sang:** Writing – review & editing, Writing – original draft. **Gang Cao:** Writing – review & editing, Supervision.

## Declaration of competing interest

The authors declare that they have no known competing financial interests or personal relationships that could have appeared to influence the work reported in this paper.
